# YAP inhibits ERα and ER^+^ breast cancer growth by disrupting a TEAD-ERα signaling axis

**DOI:** 10.1038/s41467-022-30831-5

**Published:** 2022-06-02

**Authors:** Xu Li, Shu Zhuo, Ting Zhuang, Yong Suk Cho, Guojin Wu, Yuchen Liu, Kun Mu, Kai Zhang, Peng Su, Yingzi Yang, Cheng Cheng Zhang, Jian Zhu, Jin Jiang

**Affiliations:** 1 Department of Molecular Biology, University of Texas Southwestern Medical Center, Dallas, TX 75390 USA; 2 Department of Developmental Biology, Harvard School of Dental Medicine, 188 Longwood Ave, Boston, MA 02215 USA; 3 Henan Key Laboratory of Immunology and Targeted Drugs, School of Laboratory Medicine, Xinxiang Medical University, Xinxiang, Henan 453000 PR China; 4 Department of Physiology, University of Texas Southwestern Medical Center, Dallas, TX 75390 USA; 5 Harvard Stem Cell Institute, 188 Longwood Ave, Boston, MA 02215 USA; 6 Dana-Farber/Harvard Cancer Center, 188 Longwood Ave, Boston, MA 02215 USA; 7 Department of Pathology, Qilu Hospital, Cheeloo College of Medicine, Shandong University, Jinan, Shandong 250012 PR China; 8 Department of Pathology, School of Basic Medical Sciences, Cheeloo College of Medicine, Shandong University, Jinan, Shandong 250012 PR China; 9 Department of Breast Surgery, Qilu Hospital, Cheeloo College of Medicine, Shandong University, Jinan, Shandong 250012 PR China; 10 Department of General Surgery, the Second Hospital, Cheeloo College of Medicine, Shandong University, Jinan, Shandong 250012 PR China; 11 Department of Pharmacology, University of Texas Southwestern Medical Center, Dallas, TX 75390 USA; 12 Present Address: Signet Therapeutics Inc., Research Institute of Tsinghua University In Shenzhen, Shenzhen, Guangdong, 518057 PR China

**Keywords:** Breast cancer, HIPPO signalling

## Abstract

Hippo signaling restricts tissue growth by inhibiting the transcriptional effector YAP. Here we uncover a role of Hippo signaling and a tumor suppressor function of YAP in estrogen receptor positive (ER^+^) breast cancer. We find that inhibition of Hippo/MST1/2 or activation of YAP blocks the ERα transcriptional program and ER^+^ breast cancer growth. Mechanistically, the Hippo pathway transcription factor TEAD physically interacts with ERα to increase its promoter/enhancer occupancy whereas YAP inhibits ERα/TEAD interaction, decreases ERα occupancy on its target promoters/enhancers, and promotes ERα degradation by the proteasome. Furthermore, YAP inhibits hormone-independent transcription of ERα gene (*ESR1*). Consistently, high levels of YAP correlate with good prognosis of ER^+^ breast cancer patients. Finally, we find that pharmacological inhibition of Hippo/MST1/2 impeded tumor growth driven by hormone therapy resistant ERα mutants, suggesting that targeting the Hippo-YAP-TEAD signaling axis could be a potential therapeutical strategy to overcome endocrine therapy resistance conferred by ERα mutants.

## Abstract (web summary)

Recent studies have reported that oncoprotein YAP can function as tumour suppressor in certain contexts. Here the authors show that inhibition of Hippo signalling or YAP activation blocks ERα transcriptional program and ER + breast cancer growth and mechanistically this is through YAP interfering with TEAD-ERα signalling axis.

## Introduction

The Hippo pathway is an evolutionarily conserved signaling pathway that restricts tissue growth by inhibiting cell proliferation and promoting apoptosis^1–3^. In the canonical Hippo pathway, MST1/2 (Hippo in *Drosophila*) functions as an upstream kinase that phosphorylates and promotes the activity of a downstream kinase LATS1/2^4–8^. LATS1/2 in turn phosphorylates and inhibits the activity of YAP/TAZ^9–11^. When Hippo signaling is compromised, unphosphorylated YAP/TAZ enters the nucleus and binds the pathway transcription factor TEAD1-4 to activate the expression of Hippo target genes^12–14^. Accumulating evidence suggests that mammalian Hippo signaling regulates cell contact inhibition, organ size, and tumorigenesis^15–18^. *YAP* is amplified and its protein level and nuclear localization are elevated in many human cancers including liver, lung, colon, esophageal, and cervical cancers^9,10,17,18^. Furthermore, YAP overexpression or MST1/2 knockout in mouse liver resulted in hepatocellular carcinoma^10,19–23^, leading to the prevalent view that Hippo signaling functions as a tumor suppressor pathway by blocking the oncogenic potential of YAP.

Estrogen receptor α (ERα) belongs to the ligand-dependent subfamily of the nuclear receptor superfamily of transcription factors, and its activity is regulated mainly by estrogen as well as by estrogen-independent mechanisms such as phosphorylation in response to growth factors^24^. ERα is essential for the progression of luminal types of breast cancer and it promotes tumor growth by activating a transcriptional program in a hormone-dependent manner^25^. Because of this, hormone depletion and ERα antagonists have been widely used to treat ER^+^ breast cancer patients; however, drug resistance often occurs in patients after prolonged treatment. Many different mechanisms of endocrine resistance have been identified, including mutations in the ERα gene that lead to altered receptor activity, which typically occur under therapy with aromatase inhibitors^26–28^.

In this study, we uncover a role of Hippo signaling and a tumor suppressor function of YAP in the regulation of ER^+^ breast cancer. We find that pharmacological inhibition of the MST1/2 kinase activity, as well as ectopic activation of YAP, inhibits ERα transcription program and ER^+^ breast cancer growth. Mechanistically, we find that TEAD and ERα co-occupy the promoter/enhancer regions of a large set of ERα target genes and that TEAD physically interacts with ERα to promote ERα binding to its target promoters/enhancers. When Hippo signaling is inhibited, YAP translocates to the nucleus and competes with ERα for binding to TEAD, leading to dissociation of ERα from its target sites and subsequent degradation of ERα. Consistent with these findings, high levels of YAP correlate with a longer relapse-free survival rate and good tamoxifen treatment outcome in ER^+^ breast cancer patients while high levels of MST1/2 or TEAD4 correlate with poor prognosis. Finally, we demonstrate that MST1/2 inhibition can overcome tamoxifen resistance conferred by frequently occurring point mutations in ERα, suggesting that targeting the Hippo pathway could be a potential strategy to overcome endocrine therapy resistance in breast cancer patients.

## Result

### YAP expression is low in ER^+^ breast cancer and serves as a good prognosis marker

**Fig. 1:**
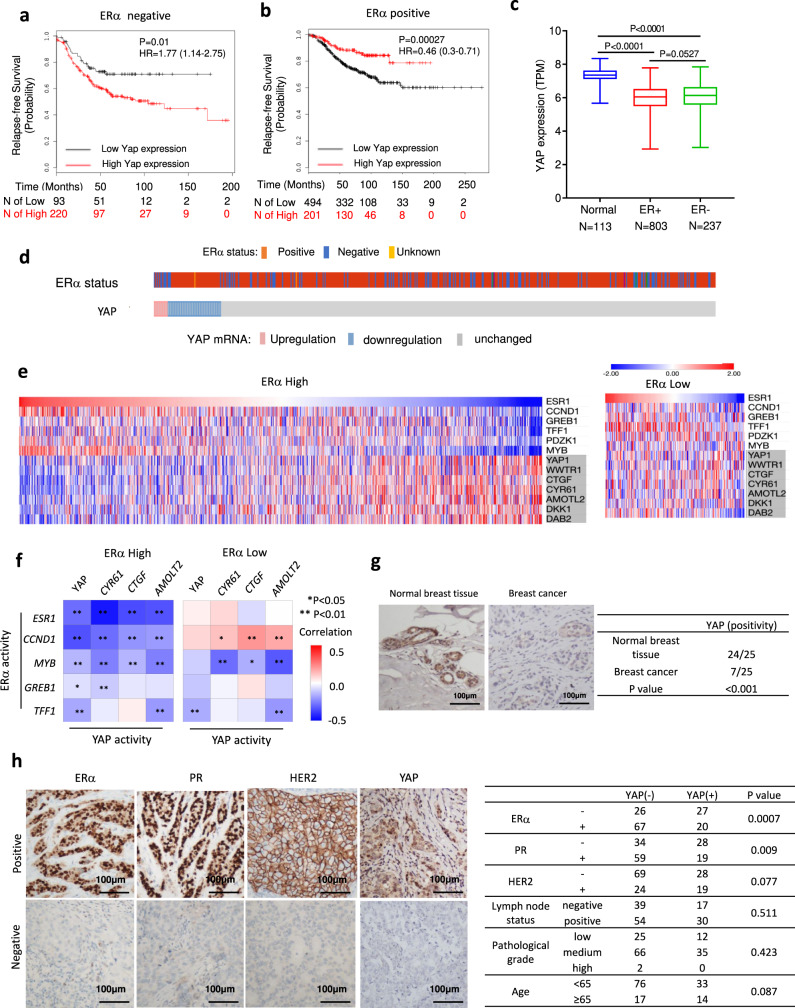
The expression level and prognostic effect of YAP in ERα-positive breast tumors. **a** Kaplan–Meier graph of relapse-free survival shows that high *YAP* relates to poor prognosis in ERα negative breast tumor. **b** Kaplan–Meier graph of relapse-free survival shows that high *YAP* favors the survival in ERα positive breast cancer patients. **c** Analysis of TCGA database shows that *YAP* mRNA level is decreased in both ER+ and ER− breast cancer samples compared with normal breast tissues. Normal group, minima = 5.669927, maxima = 8.34457571, mean = 7.280, *n* = 113, ER+ group, minima = 2.9289187, maxima = 7.78269181, mean = 5.931, *n* = 803, ER− group, minima = 3.02059779, maxima = 7.84498136, mean = 6.055, *n* = 237. *p*-value calculated by one way ANOVA. **d** *YAP* expression pattern in ERα positive and negative breast cancer cells. Data were derived from TCGA database (https://www.cbioportal.org). **e** Heatmap for the expression of ERα signaling and YAP signaling pathway target genes (shaded) in TCGA ERα high (left) and ERα low (right) breast cancer datasets (in the diagram, red represents high gene expression levels, blue represents low gene expression levels). **f** Heatmap of the correlation between ERα target genes and YAP target genes in TCGA ER high (left) and ERα low (right) breast cancer datasets. The horizontal and vertical coordinates represent genes, and different colors represent correlation coefficients (in the diagram, red represents positive correlation, blue represents negative correlation), the darker the color represents the stronger the correlation. Asterisks represent levels of significance (*p < 0.05, **p < 0.01). **g** YAP expression is decreased in human breast cancers compared with normal breast tissues in immunohistochemistry analysis (*P* < 0.001). Examples of YAP staining in normal and ER^+^ breast tumor samples were shown at ×40 magnification. Results are representative of one set of experiments. **h** YAP expression is reversely correlated with ERα/PR positivity (*P* = 0.0007; *P* = 0.009 respectively). Examples of positive/negative YAP, ERα, PR, and HER2 staining in breast tumor samples were shown at ×40 magnification. Results are representative of one set of experiments. Two-sided log-rank test were used for **a** and **b**. Chi-squared test were used for **g** and **h**.

Consistent with YAP acting as an oncoprotein, high *YAP* levels correlate with poor prognosis of ER^−^ breast cancer patients (Fig. 1a)^29^; therefore, we were surprised to find that the publicly available clinical data indicate that high *YAP* levels correlate with good prognosis of ER^+^ breast cancer (Fig. 1b). By analyzing the TCGA database, we found that *YAP* mRNA is decreased in breast cancer tissues compared with normal breast tissues (Fig. 1c). In breast cancer samples, *YAP* expression negatively correlates with ERα expression (Fig. 1d). Furthermore, analysis of 1247 TCGA breast cancer samples revealed that ERα signaling activity (represented by the expression of ERα target genes) and YAP signaling activity (indicated by the expression of YAP signature target genes) are negatively correlated in ERα high breast cancer datasets (Fig. 1e, f).

To examine the expression of YAP in breast cancer at the protein level, we analyzed the expression of YAP in breast cancer patient samples via immunohistochemistry (IHC). Compared with normal breast tissues, YAP expression was significantly decreased in breast tumors (24/25 vs 7/25; *P* < 0.001, Fig. 1g; Supplementary Data 1). In addition, we investigated the correlation between YAP expression and the breast cancer molecular markers, including ERα, PR (progesterone receptor), and HER2 (human epidermal growth factor receptor 2). In 140 breast tumor tissues analyzed via IHC, YAP expression negatively correlated with ERα/PR (*P* = 0.0007 and *P* = 0.009, respectively; Fig. 1h; Supplementary Data 2), consistent with a previous report^30^.

Consistent with its role in inhibiting YAP, high *MST1/2* expression correlates with poor prognosis in ER^+^ breast cancer patients (Supplementary Fig. 1a, b). Furthermore, like YAP, high *TAZ* expression correlates with good prognosis in ER^+^ breast cancer patients but poor prognosis in ER^−^ breast cancer patients (Supplementary Fig. 1c, d). As ER^+^ breast cancer patients are mainly treated with tamoxifen, we further analyzed *YAP* expression data in tamoxifen-treated patients. Four independent datasets indicate that high *YAP* levels correlate with longer relapse-free survival time in tamoxifen-treated patients (Supplementary Fig. 1e–h). By analyzing breast cancer in the METABRIC cohort, we found that high *YAP* expression and YAP signature correlate with good prognosis in ER^+^ breast cancer patients (Supplementary Fig. 1i, j). Taken together, these clinic data imply that YAP may function as a tumor suppressor in ER^+^ breast cancer.

### Inhibition of MST1/2 or activation of YAP blocks ER^+^ breast cancer cell growth

**Fig. 2:**
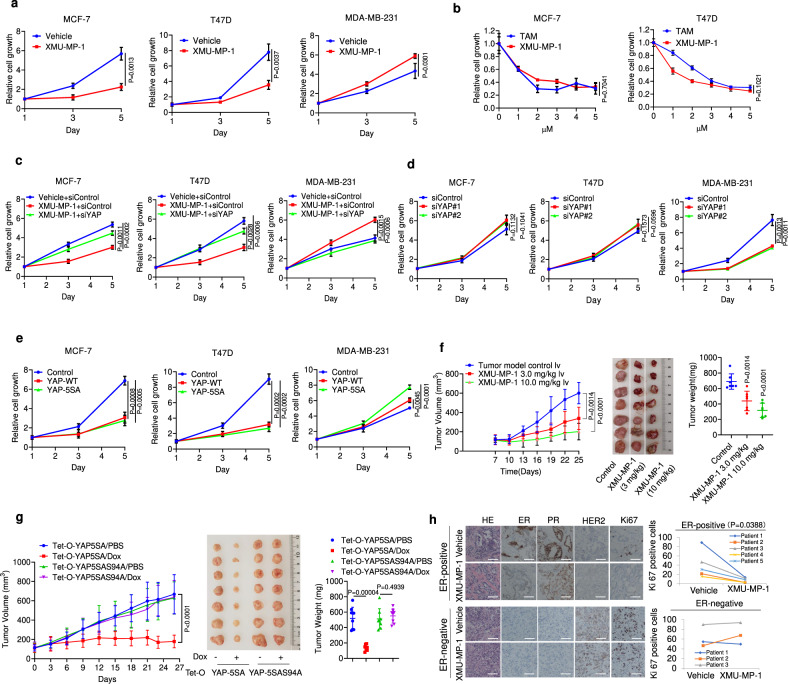
YAP inhibits ERα-positive breast cancer cell growth. **a** XMU-MP-1 inhibits MCF-7 and T47D cancer cell proliferation but facilitates MDA-MB-231 cancer cell growth. **b** Comparison of inhibitory efficacy between tamoxifen and XMU-MP-1 on MCF-7 and T47D cancer cell growth. **c** YAP siRNA blocked the inhibitory effect of XMU-MP-1 on MCF-7 and T47D cancer cell growth as well as the stimulatory effect of XMU-MP-1 on MDA-MB-231 cancer cell growth. **d** YAP depletion promoted MCF-7 and T47D but inhibited MDA-MB-231 cancer cell growth. **e** Overexpression of either wild-type YAP or YAP-5SA inhibited MCF-7 and T47D but facilitated MDA-MB-231 cancer cell growth. **f** XMU-MP-1 inhibited ER-positive breast cancer growth in xenografts. Female NOD scid gamma (NSG) mice bearing MCF-7 tumors were treated daily with vehicle or XMU-MP-1 at the indicated concentrations. Tumor growth curve (left), photograph of tumor samples (middle), and quantification of tumor weight (right) at the end of treatment were shown, each group *n* = 7. **g** YAP-5SA but not YAP-5SAS94A inhibited ER-positive breast cancer growth in xenografts bearing MCF-7 cells that stably expressed the indicated constructs. The mice were injected i.p. with PBS containing Dox (20 mg/kg) or PBS daily for the indicated period of time. Tumor growth curve (left), photograph of tumor samples (middle), and quantification of tumor weight (right) at the end of treatment were shown, each group *n* = 7. **h** XMU-MP-1 inhibited cell proliferation of ER-positive breast cancer samples but did not inhibit ER-negative breast cancer patient samples in patient-derived explant (PDEx) assay. The patient-derived tumor samples were cultured ex vivo on gelatin sponges for 48 h with 10% FBS in the presence of 3 μM XMU-MP-1 or vehicle. The tumor samples were fixed and stained with ERα, PR, HER2, and Ki67 via IHC analysis (left). The dynamic change of Ki67 positive cells were counted and shown (right). Scale bars are 100 μm. Results shown in **a**–**e** are representative of three independent experiments while results shown in **f**–**h** are based on one set of experiments. Data are means ± s.d. Two-side, unpaired *t*-test for **a**–**h**. Source data are provided in the Source Data file.

To determine whether increasing YAP activity can inhibit the growth of ER^+^ breast cancer cells, we took advantage of a pharmacological small molecule inhibitor, XMU-MP-1 (Supplementary Fig. 2a), which specifically inhibits the kinase activity of MST1/2, thereby promoting YAP nuclear localization and activation^31^. We found that XMU-MP-1 inhibited the growth of ER^+^ breast cancer cell lines including MCF-7 and T47D in a dose-dependent manner but enhanced the growth of MDA-MB-231, an ER^−^ breast cancer cell line (Fig. 2a, b). Immunostaining or fractionation of MCF-7 cells treated with XMU-MP-1 revealed an increased YAP nuclear localization (Supplementary Fig. 2b–d). Furthermore, the inhibitory effect of XMU-MP-1 on the growth of MCF-7 cells was partially reversed by siRNA-mediated YAP knockdown (KD) (Fig. 2c), suggesting that MST1/2 inhibition blocked ER^+^ breast cancer cell growth at least in part through YAP. Consistent with the notion that YAP activation inhibits ER^+^ breast cancer cells, YAP KD increased while overexpression of either wild-type (WT) or a constitutively active form of YAP (YAP-5SA)^9^ suppressed the growth of both MCF-7 and T47D cells (Fig. 2d, e). On the other hand, YAP KD inhibited while YAP overexpression promoted the growth of MDA-MB-231 (Fig. 2d, e), which is consistent with the previous studies^29,32,33^.

We next determined whether inhibition of Hippo signaling pathway or activation of YAP could inhibit ER^+^ breast cancer progression in xenograft tumor models. Indeed, daily injection of XMU-MP-1 in estrogen-supplemented female NOD scid gamma (NSG) mice bearing MCF-7 xenografts over a 3-week period significantly inhibited tumor growth compared with vehicle treatment (Fig. 2f), suggesting that MST1/2 inhibition can reduce ER^+^ breast cancer growth in vivo. Examination of xenograft tumors treated with XMU-MP-1 showed elevated YAP nuclear localization and target gene expression (Supplementary Fig. 2e, f), confirming the on-target effects of drug treatment in vivo. We also generated MCF-7 cell lines stably expressing inducible YAP-5SA (Tet-O-YAP-5SA) or its TEAD-binding defective form (Tet-O-YAP-5SAS94A)^14^ and derived xenografts carrying these cell lines. Dox-induced expression of YAP-5SA dramatically reduced tumor growth while expression of YAP-5SAS94A had no effect (Fig. 2g), suggesting that YAP activation suppresses ER^+^ breast cancer growth in vivo and does so through binding to TEAD.

We also utilized an ex vivo culture model of primary breast tumors derived from patients, which allows the evaluation of drugs on breast tumors that maintain their native tissue architecture^34,35^. Surgically extirpated breast tumor samples were sliced into small pieces and grown ex vivo for 48 h on a gelatin sponge in the presence or absence of 2 μM XMU-MP-1. We found that incubation of XMU-MP-1 with ER^+^ breast tumor samples dramatically decreased their proliferation compared to vehicle-treated controls (*n* = 5; Fig. 2h; Supplementary Fig. 2g, h). Furthermore, XMU-MP-1 treatment significantly reduced the ER/PR expression in the ER^+^ tumors (Fig. 2h; Supplementary Fig. 2g, h). Importantly, XMU-MP-1 treatment had little if any effect on the proliferation of ER-negative breast tumors (*n* = 3; Fig. 2h), suggesting that XMU-MP-1 selectively inhibits the proliferation of patient-derived ER^+^ breast tumors.

### Inhibition of MST1/2 or activation of YAP inhibits ERα transcriptional program

**Fig. 3:**
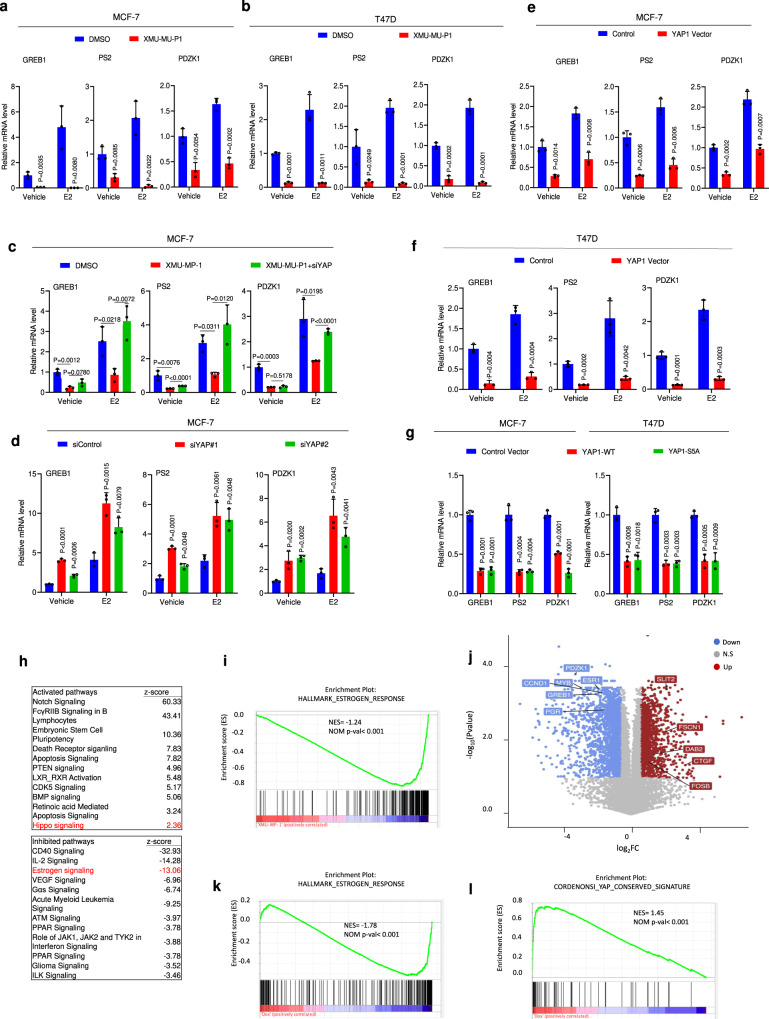
YAP suppresses ERα signaling activity in breast cancer cells. **a, b** XMU-MP-1 inhibited ERα target gene expression in MCF-7 (**a**) and T47D (**b**) cells. Cells grown in hormone deletion medium were treated for 3 μM XMU-MP-1 for 8 h and then treated with 10 nM 17β-Estradiol (E2) or vehicle for 6 h, followed by RT-qPCR analysis of *GREB1*, *PS2*, and *PDZK1* expression. **c** YAP depletion rescued ERα target gene expression inhibited by XMU-MP-1. MCF-7 cells were transfected with control or YAP siRNA for 48 h in hormone deletion medium. The cells were treated for 3 μM XMU-MP-1 for 8 h and then treated with 10 nM E2 or vehicle for 6 h, followed by RT-qPCR. **d** YAP depletion increased ERα target gene expression. MCF-7 cells were transfected with control or YAP siRNA for 48 h under hormone depletion condition and then treated with 10 nM E2 or vehicle for 6 h, followed by RT-qPCR. **e**, **f** Ectopic expression of YAP inhibited ERα target gene expression. MCF-7 (**e**) or T47D (**f**) cells were stably infected with YAP or control virus. After 48 h, cells were treated with 10 nM E2 or vehicle for 6 h, followed by RT-qPCR. **g** Ectopic expression of YAP or YAP-5SA inhibited ERα target gene expression. MCF-7 or T47D cells were stably infected with wild-type YAP, YAP-5SA, or control virus. After 48 h, the total RNA was extracted for RT-qPCR analysis of *GREB1*, *PS2* and *PDZK1* expression. **h** Top 10 signaling pathways significantly decreased (top) or increased (bottom) in MCF-7 cells treated with XMU-MP-1. The cells were treated with vehicle or 3 μM XMU-MP-1 for 8 h. Threshold *P* < 0.001 and fold change > 2. *n* = 3. **i** Gene set enrichment analysis (GSEA) shows a depletion of estrogen-responsive signature genes in MCF-7 cells treated with XMU-MP-1. **j** Volcano plot shows the opposite effects of XMU-MP-1 treatment on estrogen-responsive signature genes (blue) and the Hippo pathway signature genes (red) in MCF-7 cells. Threshold *P* < 0.05 and fold change > 1.5. **k**, **l** GSEA shows a depletion of estrogen-responsive signature genes (**k**) and enrichment of YAP signature genes (**l**) in MCF-7 cells expressing an inducible YAP-5SA. Results shown in **a**–**g** are representative of three independent experiments. Data are means ± s.d. Two-side, unpaired *t*-test for **a**–**g**. Source data are provided in the Source Data file.

Because the ERα transcriptional program is essential for the growth of ER^+^ breast cancer cells, we next examined the effect of MST1/2 inhibition on ERα target gene expression. RT-qPCR assay shows that treating MCF-7 or T47D cells with XMU-MP-1 decreased both the basal and estrogen (E2)-induced expression of multiple ERα target genes including *GREB1*, *PS2* (also called *TFF1*), *PDZK1,* and *CCND1* (Fig. 3a, b; Supplementary Fig. 3a, b)^36^. YAP KD by two independent siRNAs increased ERα target gene expression and alleviated the inhibitory effect of XMU-MP-1 (Fig. 3c, d; Supplementary Fig. 3c–e). On the other hand, overexpression of either YAP-WT or YAP-5SA inhibited ERα target gene expression (Fig. 3e–g).

**Fig. 4:**
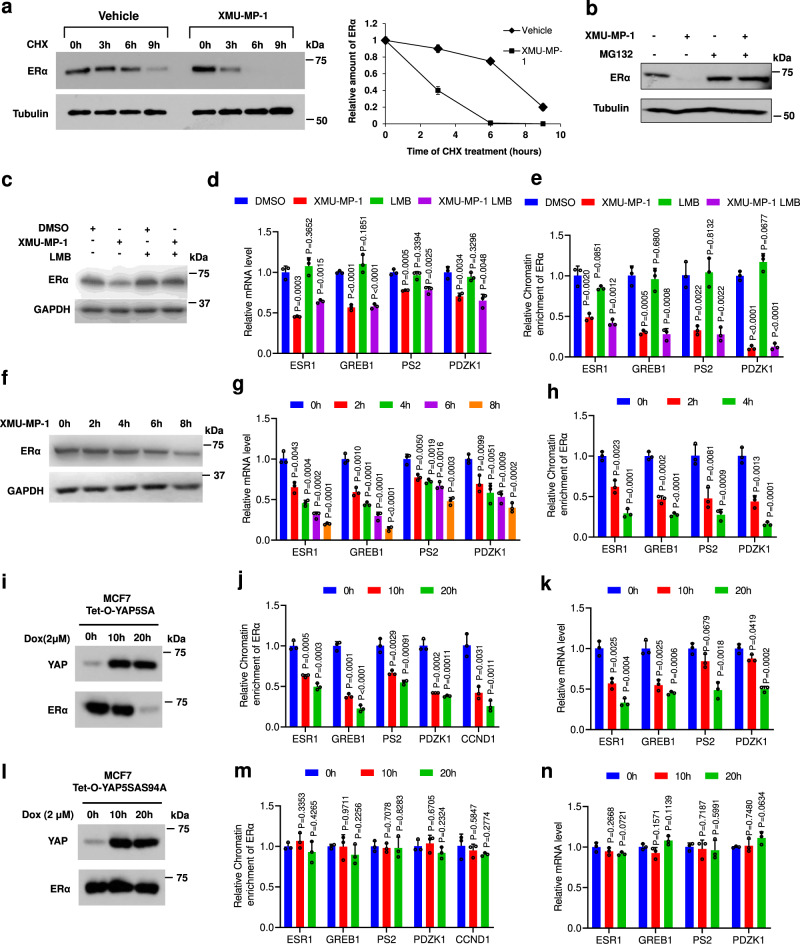
XMU-MP-1 and YAP inhibits ERα binding to its target gene promoters/enhancers. **a** XMU-MP-1 decreased the half-life of ERα. MCF-7 cells were treated with vehicle or 3 μM XMU-MP-1 in the presence of cycloheximide for the indicated times, followed by western blot analysis. **b** XMU-MP-1 downregulated ERα through proteosome. MCF-7 cells were treated with vehicle or 3 μM XMU-MP-1 in the absence or presence of 10 μM MG132, followed by western blot analysis with the indicated antibodies. **c** XMU-MP-1-promoted ERα degradation was inhibited by Leptomycin B (LMB). MCF-7 cells were treated with vehicle or 3 μM XMU-MP-1 in the absence or presence of 50 nM LMB, followed by western blot analysis. **d**, **e** XMU-MP-1 inhibited ERα target gene expression (**d**), inhibited ERα binding to its target gene promoters/enhancers (**e**) even when ERα degradation was inhibited by LMB. MCF-7 cells were treated with vehicle or 3 μM XMU-MP-1 in the absence or presence of 50 nM LMB, followed by RT-qPCR analysis of *GREB1*, *PS2*, *PDZK1*, and *ESR1* expression (**d**) and ChIP-qPCR analysis for ERα binding to the promoter/enhancer regions of *PS2*, *GREB1*, and *PDZK1*. **f**–**h** Time course experiments showed that XMU-MP-1 inhibited ERα transcriptional activity and binding to its target promoters/enhancers prior to ERα protein downregulation. MCF-7 cells were treated with 3 μM XMU-MP-1 for the indicated time, followed by western blot analysis for ERα (**f**), RT-qPCR analysis for ERα target gene expression (**g**), and ChIP-qPCR analysis for ERα binding to the promoter/enhancer regions of its target genes (**h**). **i**–**n** Induced expression of YAP-5SA but not YAP-5SAS94A blocked ERα transcriptional activity and binding to its target promoters/enhancers. MCF-7 cells expressing *Tet-O-YAP5SA* (**i**–**k**) or *Tet-O-YAP5SAS94A* (**I**–**n**) were treated with 0.2 μg/ml doxycycline (Dox) for the indicated time, followed by western blot analysis for YAP1 and ERα (**i**, **l**), ChIP-qPCR analysis for ERα binding to its target gene promoter/enhancer regions (**j**, **m**), and RT-qPCR analysis for ERα target gene expression (**k**, **n**). Results shown in **a**–**n** are representative of three independent experiments. Data are means ± s.d. Two-sided, unpaired *t*-test for **d**, **e**, **g**, **h**, **j**, **k**, **m**, **n**. Source data are provided in the Source Data file.

To obtain a global view of how Hippo pathway inhibition affects the ERα transcriptome, we carried out RNA-seq experiments of MCF-7 cells treated with XMU-MP-1 or vehicle. Gene Ontology (GO) enrichment analysis of differentially expressed genes revealed that ERα transcriptome is among the top downregulated transcriptional programs while Hippo/YAP pathway target genes are among the top upregulated transcriptional programs (Fig. 3h). Gene Set Enrichment Analysis (GSEA) revealed enrichment of estrogen-responsive genes that were downregulated by XMU-MP-1(Fig. 3i). *ESR1*, which encodes ERα, was also downregulated by XMU-MP-1, because ERα can auto-regulate its own transcription (Fig. 3j)^37–39^, and in addition, YAP can inhibit *ESR1* transcription (see below)^40^. Indeed, RT-qPCR analysis confirmed that both XUM-MP1 and YAP-5SA inhibited *ESR1* expression (see Fig. 4d, j). GSEA analysis of a recently published RNA-seq dataset revealed that estrogen-responsive genes were enriched in the downregulated while YAP-responsive signature genes in the upregulated genes in MCF-7 cells expressing an inducible YAP-5SA (Fig. 3k, l)^41^.

### YAP inhibits ERα binding to its target promoter/enhancer and promotes ERα degradation

Western blot analysis revealed that XMU-MP-1 downregulated ERα protein levels in both MCF-7 and T47D cells in a dose-dependent manner (Supplementary Fig. 4a, b). On the other hand, YAP KD increased ERα protein levels and reversed the effect of XMU-MP-1 (Supplementary Fig. 4c–e). Overexpression of YAP-WT or YAP-5SA also downregulated ERα protein in both MCF-7 and T47D cells (Supplementary Fig. 4f–g).

To determine whether Hippo signaling regulates ERα at the post-translational level, we blocked protein synthesis with cycloheximide and found that XUM-MP-1 reduced the half-life of ERα protein (Fig. 4a). Treating cells with a proteasome inhibitor MG132 prevented the downregulation of ERα protein induced by XUM-MP-1 (Fig. 4b). Treating cells with the nuclear export inhibitor leptomycin (LMB) also restored ERα protein levels in MCF-7 cells treated with XUM-MP-1 (Fig. 4c), suggesting that ERα was exported to the cytoplasm and degraded by the ubiquitin/proteosome (UPS) system. However, LMB treatment failed to rescue the downregulation of ERα-mediated transcription by XUM-MP-1 even though the level of ERα protein was largely rescued (Fig. 4c, d), suggesting that ERα protein degradation is not the primary cause of XUM-MP-1-mediated inhibition of ERα transcriptional program.

To determine whether Hippo signaling regulates ERα transcriptional activity, we asked whether XUM-MP-1 interfered with ERα binding to the promoter/enhancer regions of its target genes. ChIP-qPCR experiments revealed that XUM-MP-1 inhibited the promoter/enhancer occupancy of ERα on multiple target genes even when ERα degradation was blocked by LMB (Fig. 4e). Furthermore, treating MCF-7 cells with XUM-MP-1 for shorter periods of time (2 or 4 h) reduced ERα binding to its target promoters/enhancers as well as ERα target gene expression without notably affecting ERα protein levels (Fig. 4f–h). Similarly, doxycycline-induced expression of YAP5SA for 10 h also inhibited ERα binding to its target promoters/enhancers and ERα target gene expression without affecting ERα protein levels (Fig. 4i–k). These data suggest that dissociation of ERα from its target promoters/enhancers precedes its protein degradation. Finally, doxycycline-induced expression of YAP5SAS94A neither inhibited ERα binding to its target promoters/enhancers nor suppressed ERα target gene expression (Fig. 4l–n), suggesting that YAP inhibits ERα binding to its target promoters/enhancers through interacting with TEAD.

### TEAD is required for ERα transcriptional activity and ER^+^ breast cancer cell growth

**Fig. 5:**
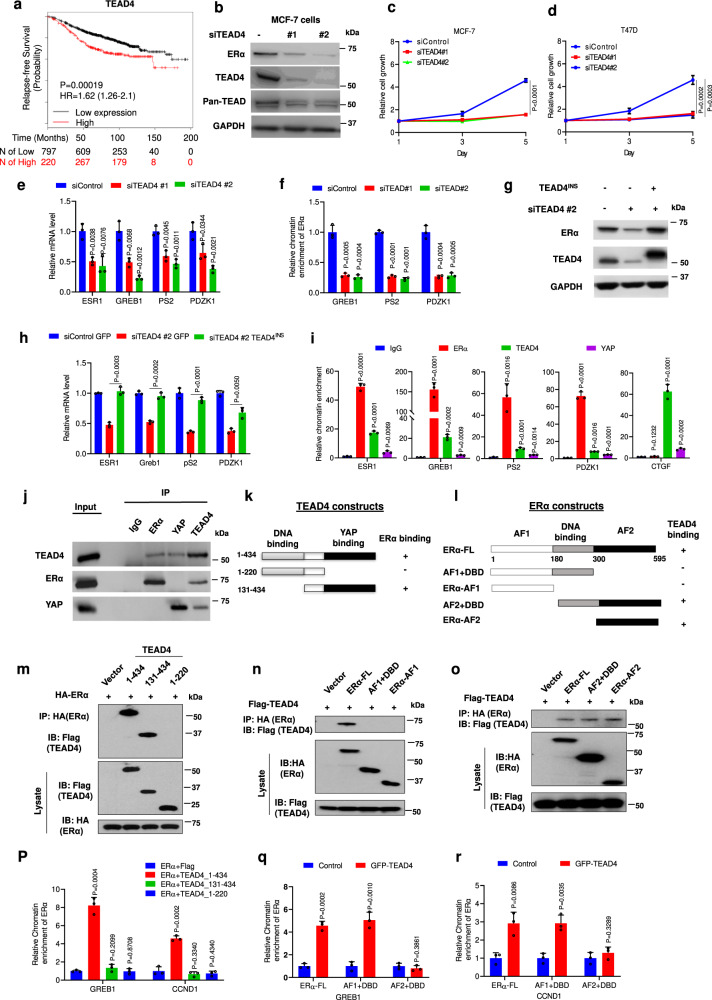
TEAD interacts with ERα and promotes ERα signaling activity. **a** High levels of *TEAD4* correlate with poor survival in endocrine therapy breast cancer patients based on https://kmplot.com/analysis/. **b** The effect of TEAD4 RNAi on TEAD4 and ERα expression in MCF-7. **c**, **d** TEAD4 RNAi inhibited ER+ breast cancer cell growth. MCF-7 (**c**) and T47D (**d**) cells transfected with control or TEAD4 siRNA were analyzed using the WST-1 assay at indicated time points. **e** TEAD4 depletion reduced ERα target gene expression. MCF-7 cells transfected with TEAD4 siRNA were grown for 48 h, followed by RT-qPCR analysis of the indicated ERα target genes. **f** ChIP-qPCR assay showed that TEAD4 RNAi decreased ERα recruitment to its target gene promoters/enhancers. **g** Western blot analysis of ERα and TEAD4 expression in control or MCF-7 cells stably expressing RNAi-insensitive TEAD4 (TEAD4^INS^) and transfected with control or TEAD4 siRNA. **h** TEAD4^INS^ restored the expression of ERα target genes in TEAD4 depleted MCF-7 cells. **i** ChIP-qPCR assay showed that TEAD4 and ERα co-occupied on ERα target promoters/enhancers in MCF-7 cells. **j** Co-IP experiments revealed that TEAD4 formed a complex with ERα in MCF-7 cells. **k**, **l** TEAD4 (**k**) or ERα (**l**) domain structure and deletion mutants used for Co-IP experiments. **m**–**o** TEAD4 interacted with the AF2 domain of ERα through its YAP-binding domain. HEK293T cells were transfected with the indicated TEAD4 and ERα constructs, followed by Co-IP and western blot analyses. **p** ERα recruitment to its target gene promoters/enhancers is enhanced by TEAD4_1-434_ but not by TEAD4 deletion mutants TEAD4_1-220_ or TEAD4_131-434_. HEK293T cells were transfected with HA-ERα together and the indicated TEAD4 constructs, followed by ChIP-qPCR analysis of HA-ERα binding to ERα target promoters/enhancers. **q**, **r** AF2 is required for TEAD4 to facilitate ERα binding to its target promoters/enhancers. HEK293T cells were transfected with GFP-TEAD4 and the indicated ERα constructs, followed by ChIP-qPCR analysis of occupancy of ERα or its deletion mutants on ERα target promoters/enhancers. Two-sided log-rank test were used for **a**. Results shown in **b**–**j**, **m**–**r** are representative of three independent experiments. Data are means ± s.d. Two-sided, unpaired *t*-test for **c**–**f**, **h**, **i**, **p**–**r**. Source data are provided in the Source Data file.

YAP acts through the TEAD family of transcription factors to regulate the expression of Hippo pathway target genes^12–14^. Indeed, the TEAD-binding defective form of YAP (YAP-5SAS94A) failed to inhibit ER^+^ breast cancer cell growth or downregulate ERα target genes (Figs. 2g; 4l–n). Therefore, we were surprised to find that high levels of TEAD4 correlated with poor prognosis in ER^+^ breast cancer patients (Fig. 5a), which is in contrast to YAP (Fig. 1b), implying that TEAD may promote ER^+^ breast cancer progression. Consistent with the clinic data, we found that siRNA-mediated depletion of TEAD4 downregulated ERα target gene expression and inhibited ER^+^ breast cancer cell growth (Fig. 5b–e). TEAD4 KD also downregulated *ESR1* expression, ERα protein level, and ERα binding to its target promoters/enhancers (Fig. 5b, e, f). The expression of an RNAi-insensitive TEAD4 (TEAD4^INS^) rescued the downregulation of ERα target gene expression caused by TEAD4 KD (Fig. 5g, h). Knockdown of TEAD1, TEAD2, or TEAD1/3/4 also inhibited ERα target gene expression (Supplementary Fig. 5a, b, d–g), suggesting that multiple TEAD family members may act additively to regulate ERα target gene expression.

### TEAD physically interacts with ERα and promotes ERα binding to its target promoters/enhancers

To determine whether TEAD regulates ERα binding to its target promoters/enhancers precedes the downregulation of protein ERα level, we knocked down TEAD4 in MCF-7 cells under hormone depletion conditions in which inhibition of ERα binding to chromatin does not affect its stability^42^. Control and TEAD4 RNAi cells were stimulated with E2 for 3 h, followed by western blot, RT-qPCR, and ChIP-qPCR analyses. We found that TEAD4 KD reduced ERα occupancy on its target promoters/enhancers and inhibited ERα target gene expression without affecting ERα protein level (Supplementary Fig. 5h–j).

By ChIP-qPCR, we found that TEAD4 co-occupied with ERα on the promoter/enhancer regions of ERα target genes (Fig. 5i). YAP also occupied the same regions on ERα target promoters/enhancers albeit less effectively compared with its binding to the *CTGF* promoter/enhancer (Fig. 5i). To obtain a global view of how ERα, TEAD, YAP bind chromatin in MCF-7 cells, we analyzed previously published ChIP-seq datasets^41,43^. We found that ERα and TEAD1 co-bound 10,848 peaks on chromatin, a fraction of which (3040) were also bound by YAP (Supplementary Fig. 6a–c). GSEA analysis indicated that the ERα/TEAD1 co-bound genes were enriched among XMU-MP-1 or YAP-5SA downregulated genes in MCF-7 cells (Supplementary Fig. 6d, e). Among the 165 XMU-MP-1 downregulated ERα target genes that contain ER binding peaks in their promoter/enhancer regions, 139 (84%) contain ERα/TEAD co-binding peaks whereas 26 (16%) contain ER only peaks (Supplementary Fig. 6f, g; Supplementary Data 3). Hence, TEAD and ERα may cooperate to regulate a large fraction of ERα target genes.

Co-IP experiments revealed that TEAD4, as well as TEAD1, formed a complex with ERα (Fig. 5j; Supplementary Fig. 5c). By contrast, YAP did not form a complex with ERα although YAP interacted with TEAD4 in the same Co-IP experiments (Fig. 5j), suggesting that ERα/TEAD and YAP/TEAD exist in separated complexes. To determine whether the formation of the TEAD/ERα complex on chromatin facilitates ERα binding to its target promoters/enhancers, we mapped the domains in TEAD4 and ERα that mediate their interaction in HEK293T cells. TEAD4 protein contains an N-terminal TEA domain that interacts with DNA and a C-terminal YAP-binding domain (Fig. 5k). ERα protein consists of AF1 domain (aa1–180) that binds ERα cofactors, DNA-binding domain (aa181–300) that binds ER-responsive elements (ERE), and AF2 domain (aa300–595) that binds ligand (Fig. 5l). Deletion analysis indicated that TEAD4 interacted with ERα through its YAP-binding domain whereas ERα interacted with TEAD4 through its AF2 domain (Fig. 5m–o). ChIP-qPCR experiments showed that expression of full-length TEAD4 (1–434) enhanced the binding of co-expressed ERα to its target promoters/enhancers in HEK293T cells but an expression of TEAD4 deletion mutants lacking either the DNA-binding domain (TEAD4_131–434) or ERα-binding domain (TEAD4_1–220) failed to do so (Fig. 5p). In addition, deletion of the TEAD-binding domain from ERα (ERα_AF1 + DBD) also abolished its ability to bind cooperativity with TEAD4 on its target promoters/enhancers (Fig. 5q, r).

### YAP interferes with ERα-TEAD cooperativity by competing for TEAD

**Fig. 6:**
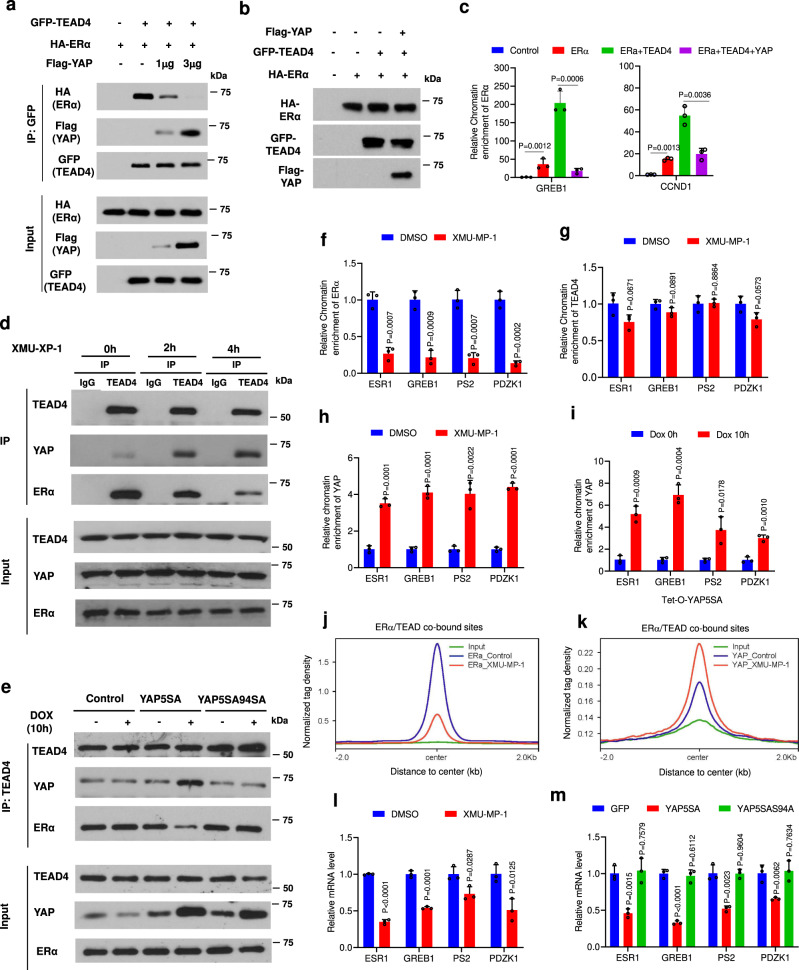
YAP inhibits ERα-TEAD interaction and hormone-independent *ESR1* expression. **a** Coexpression of YAP with ERα and TEAD4 disrupted their association. HEK293T cells were transfected with fixed amounts of GFP-TEAD4 and HA- ERα and increasing amounts of Flag-YAP, followed by Co-IP and western blot analyses. **b**, **c** YAP inhibited TEAD4-mediated enhancement of ERα binding to its target promoters/enhancers. HEK293T cells were transfected with the indicated constructs, followed by western blot analysis (**b**) or ChIP-qPCR analysis for HA-ERα binding to its target promoters/enhancers (**c**). **d** XUM-MP-1 dissociated ERα from TEAD4 while promoted YAP/TEAD4 association. MCF-7 cells were treated with 2 μM XMU-MP-1 for the indicated time, followed by Co-IP and western blot analyses. **e** YAP inhibited ERα-TEAD4 association by binding to TEAD4. Control or MCF-7 cells expressing *Tet-O-YAP5SA* or *Tet-O-YAP5SAS94A* were treated with 0.2 μg/ml Dox for 10 h, followed by Co-IP and western blot analyses. **f**–**h** XUM-MP-1 increased the promoter/enhancer occupancy of YAP to ERα target genes including *ESR1*. MCF-7 cells were treated with 3 μM XMU-MP-1 for 6 h, followed by ChIP-qPCR analysis for ERα (**f**), TEAD4 (**g**), YAP (**h**) binding to ERα target promoters/enhancers. **i** Ectopic expression of active form of YAP increased YAP binding to ERα target promoters/enhancers. MCF-7 cells expressing *Tet-O-YAP5SA* were treated with 0.2 μg/ml Dox for the indicated time, followed by ChIP-qPCR analysis for YAP binding to ERα target promoters/enhancers. **j**, **k** Aggregate plots showing the normalized tag density of ERα (**j**) and YAP1 (**k**) ChIP-seq data for the ERα/TEAD sites in MCF-7 cells treated with vehicle or XMU-MP-1. **l** XUM-MP-1 inhibited hormone-independent transcription of *ESR1* and ERα target genes. MCF-7 cells grown in hormone-depleted medium were treated 3 μM XMU-MP-1 for 6 h, followed by RT-qPCR analysis of the indicated genes. **m** YAP5SA but not YAP5SAS94A inhibited hormone-independent transcription of *ESR1* and other ERα target genes. Control or MCF-7 cells expressing *Tet-O-YAP5SA* or *Tet-O-YAP5SAS94A* were treated with 0.2 μg/ml Dox for 16 h, followed by RT-qPCR analysis of the indicated genes. Results shown in **a**–**i** and **l**, **m** are representative of three independent experiments. Data are means ± s.d. Two-sided, unpaired *t*-test for **c**, **f**–**i**, **l**, **m**. Source data are provided in the Source Data file.

When co-expressed in HEK293T cells, GFP-TEAD4 formed a complex with HA-ERα and facilitated ERα binding to its target promoters/enhancers (Fig. 6a–c). Coexpression of Flag-YAP decreased the amount of HA-ERα pulled down by GFP-TEAD4 with a concomitant increase of YAP-TEAD4 complex formation (Fig. 6a). In addition, coexpression of Flag-YAP blocked the ability of GFP-TEAD4 to promote HA-ERα binding to its target promoters/enhancers in HEK293T cells (Fig. 6b, c). Treating MCF-7 cells with XMU-MP-1 increased the association between endogenous TEAD4 and YAP but decreased the association between endogenous TEAD4 with ERα (Fig. 6d). Furthermore, Dox-induced YAP5SA but not YAP5SAS94A decreased the association of ERα with TEAD4 (Fig. 6e), suggesting that binding of YAP to TEAD blocks ERα/TEAD interaction. Although treating MCF-7 cells with XMU-MP-1 for 6 h dissociated ERα from its target promoters/enhancers (Figs. 4h; 6f; Supplementary Fig. 7), TEAD4 binding to ERα target promoters/enhancers remained relatively unchanged (Fig. 6g; Supplementary Fig. 7). By contrast, XMU-MP-1 increased the binding of YAP to the promoter/enhancer regions of multiple ERα target genes including *ESR1* (Fig. 6h; Supplementary Fig. 7). Similarly, Dox-induced expression of YAP5SA for 10 h increased YAP binding while blocking ERα binding to ERα target promoters/enhancers (Figs. 4j; 6i). Furthermore, ChIP-seq experiments revealed that XMU-MP-1 reduced the global binding of ERα to ERα/TEAD co-bound sites but increased YAP binding to these sites (Fig. 6j, k; Supplementary Fig. 8). These observations suggest that YAP-TEAD and ERα-TEAD may exist in distinct complexes that bind competitively to the promoter/enhancer regions of ERα target genes including *ESR1*. MST1/2 inhibition or YAP overexpression promotes a switch of chromatin occupancy from ERα-TEAD to YAP-TEAD complexes on the promoter/enhancer regions of ERα target genes. Consistent with this competition model, we found that overexpression of VGLL4, which competes with YAP for TEAD binding^44^, also blocked ERα binding to TEAD4 in HEK293T cells, and inhibited ERα target gene expression as well as ERα protein level in MCF-7 cells (Supplementary Fig. 9).

FOXA1 is a pioneering factor required for ERα binding to its target loci by regulating chromatin accessibility^45^. To address whether FOXA1 affects YAP/TEAD chromatin binding to ERα target promoters/enhancers, we carried out ChIP experiments to examine YAP/TEAD binding to multiple ERα target promoters/enhancers after FOXA1 depletion by shRNA. We found that knockdown of FOXA1 diminished TEAD chromatin binding to ERα target promoters/enhancers in untreated cells as well as YAP/TEAD chromatin binding to multiple ERα target promoters/enhancers in MCF-7 cells treated with XMU-MP-1 (Supplementary Fig. 10), consistent with the notion that FOXA1 regulates the chromatin accessibility of ERα target loci.

### YAP inhibits hormone-independent *ESR1* transcription

The observation that XMU-MP-1 or YAP overexpression inhibits the basal expression of ERα target genes under hormone depletion conditions implies that YAP binding to ERα target promoters/enhancers may actively repress gene expression in addition to blocking ERα recruitment (Fig. 3a, b, e, f). To determine whether YAP also inhibits the basal expression of *ESR1*, MCF-7 cells grown under hormone depletion conditions were treated with XMU-MP-1, followed by RT-PCR to determine the expression of *ESR1* and other ERα target genes. We found that XMU-MP-1 treatment could inhibit the basal transcription of *ESR1* in addition to *GREB1*, *PS2*, and *PDZK1* (Fig. 6l). Similarly, expression of YAP-5SA but not YAP-5SAS94A in MCF-7 cells grown under hormone depletion conditions also inhibited the basal expression of *ESR1*, *GREB1*, *PS2*, and *PDZK1* (Fig. 6m). These results suggest that YAP inhibits the expression of *ESR1* and other ERα target genes both by inhibiting ERα binding to their promoter/promoter regions and by blocking their basal transcription, likely through recruiting co-repressors^46,47^. Consistent with our findings, a recent study showed that LATS1/2 KO inhibited *ESR1* gene expression and ER^+^ cancer cell growth through YAP/TAZ^40^.

### MST1/2 inhibition can overcome hormonal therapy resistance

**Fig. 7:**
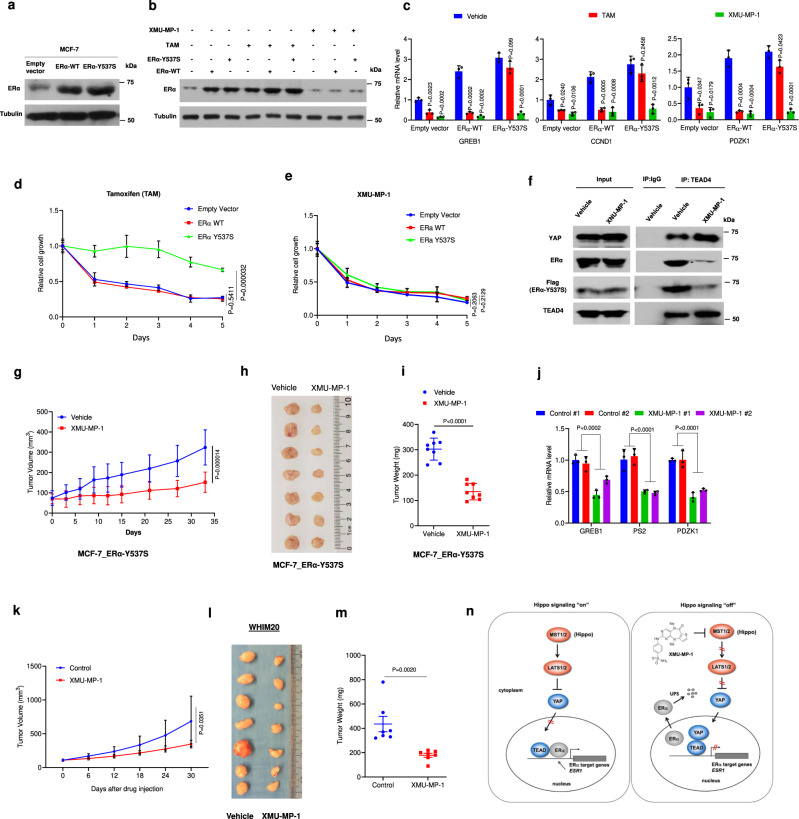
MST1/2 inhibition overcomes hormone therapy resistance. **a** Western blot analysis to show the expression ERα in control and MCF-7 cells transfected with lentivirus carrying wild-type (WT) or mutant (Y537S) ERα. **b** XMU-MP-1 downregulated both ERα-WT and ERα-Y537S. MCF-7 cells expressing ERα-WT or ERα-Y537S were treated with 3 μM XMU-MP-1 or 1 μM tamoxifen for 24 h, followed by western blot analysis. **c** XMU-MP-1 but not tamoxifen (TAM) suppressed ERα-Y537S activity. Control and MCF-7 cells expressing ERα-WT or ERα-Y537S were treated with 3 μM XMU-MP-1 or 1 μM tamoxifen for 24 h, followed by RT-qPCR analysis of ERα target gene expression. **d**, **e** XMU-MP-1 but not tamoxifen inhibited the growth of ERα-Y537S-expressing cells. MCF-7 cells expressing ERα-WT or ERα-Y537S were seeded into 96-well plates and treated with tamoxifen (**d**) or XMU-MP-1 (**e**) at the indicated concentrations, followed by the WST-1 assay. **f** XMU-MP-1 inhibited TEAD4/ERα-Y537S interaction. MCF-7 cells expressing ERα-Y537S were treated with 3 μM XMU-MP-1 for 2 h, followed by Co-IP and western blot analysis with the indicated antibodies. **g**–**j** XMU-MP-1 inhibited ERα-Y537S-expressing breast cancer tumor growth in xenograft. Female NSG mice carrying ERα-Y537S-expressing MCF-7 tumors were treated daily with vehicle or 3 mg/kg XMU-MP-1 for the indicated time. Tumor growth curve (**g**), photograph of tumor samples (**h**), quantification of tumor weight (**i**) and analysis of ERα target gene expression (**j**) at the end of treatment were shown. *n* = 7 for **g**, **h**. **k**–**m**, XMU-MP-1 inhibited hormone therapy-resistant breast cancer tumor in patient-derived xenograft model. Female NSG mice carrying WHIM20 tumors were treated daily with vehicle or 3 mg/kg XMU-MP-1 for the indicated time. Tumor growth curve (**k**), photograph of tumor samples (**l**), and quantification of tumor weight (**m**) at the end of treatment were shown. Each group *n* = 7. **n** YAP and TEAD have opposite effects on ERα transcriptional activity. See text for details. Results shown in **b**–**f** are representative of three independent experiments, results shown in **g**–**m** are from one set of experiments. Data are means ± s.d. Two-sided, unpaired *t*-test for **c**–**e**, **g**, **i**, **j**, **k**, **m**. Source data are provided in the Source Data file.

Mutations in the ligand-binding domain of ERα including Y537C/S/N and D538G are hot spots in ER^+^ cancer patients who developed resistance to hormonal therapy^27^. Among these mutations, Y537S is the most common one found in tamoxifen-resistant patients. We utilized the lentivirus system to generate MCF-7 cell lines stably expressing either the wild-type (ERα-WT) or mutant ERα (ERα-Y537S) (Fig. 7a). As expected, ERα-Y537S-expressing cells exhibited resistance to tamoxifen treatment with respect to cell proliferation and ERα target gene expression; however, ERα-Y537S and ERα-WT expressing cells were similarly sensitive to XMU-MP-1 treatment (Fig. 7b–e). Furthermore, XMU-MP-1 inhibited the interaction between TEAD4 and ERα-Y537S (Fig. 7f), which could explain why XMU-MP-1 could inhibit the transcription activity of this tamoxifen-resistant ERα mutant. Similar results were obtained with the D538G mutant (Supplementary Fig. 11).

To determine whether MST1/2 inhibition could inhibit the hormonal therapy-resistant ERα mutant in vivo, we generated ERα-Y537S-expressing MCF-7 xenografts as well as patient-derived xenograft (PDX) models bearing WHIM20 breast cancer cells. WHIM20 is a well-characterized, HER2^−^, ER^+^, PR^+^ PDX model of breast cancer^48^. Derived from a skin metastasis, this line possesses a Y537S mutation in ERα, a C182X mutation in P53, and an E542K mutation in PIK3CA, and grows independently of estradiol^48^. In both cases, daily treatment with XMU-MP-1 for up to a month significantly impaired tumor growth (Fig. 7g–l), suggesting that targeting the Hippo pathway could overcome therapy resistance conferred by the mutant ERα.

## Discussion

The Hippo signaling pathway plays an essential role in the regulation of tissue growth, stem cell activity, cancer progression, and drug resistance. Despite the prevailing view that Hippo signaling pathway functions as a tumor suppressor pathway and YAP as an oncoprotein, recent studies have implicated YAP functioning as a tumor suppressor in certain contexts. For example, YAP mediated DNA damage-induced cell death in hematological cancers by binding to P73 to activate genes involved in apoptosis^49^. Loss of Lats1/2 inhibits colorectal cancer progression through YAP-mediated inhibition of Wnt signaling and intestinal stem cell activity^50,51^. Here we have demonstrated that inhibition of Hippo signaling/activation of YAP inhibited ERα transcriptional activity and the growth of ER^+^ breast cancer growth. In addition, we have uncovered a new mechanism by which YAP acts through TEAD to regulate cancer cell growth. In the conventional model, YAP binds TEAD to either activate oncogenic transcriptional programs or inhibit the transcription of tumor suppressor genes^32,46^. Here, we showed that TEAD is a critical cofactor for ERα to activate the ERα oncogenic transcription program. TEAD physically interacts with ERα and promotes ERα binding to its target promoters/enhancers. YAP binding to TEAD disrupts the interaction between TEAD and ERα, leading to ERα dissociation from its target promoters/enhancers, followed by nuclear export and degradation by UPS (Fig. 7m).

A recent study suggested that YAP and TEAD form a trimeric complex with ERα to promote the recruitment of ERα coactivators, thus facilitating ERα target gene expression^52^. However, our Co-IP experiments clearly demonstrated that YAP and ERα competed for binding to the same pool of TEAD and that YAP/TEAD and ERα/TEAD existed in distinct protein complexes. Although ChIP experiments revealed that YAP, TEAD, and ERα “co-occupy” the promoter/enhancer regions of many ERα target genes at the population level, our results suggest that at a given chromatin binding site, YAP/TEAD and ERα/TEAD binding are mutually exclusive. In addition, our study suggests that while the binding of ERα/TEAD to ERα target promoter/enhancer regions recruits coactivators, the binding of YAP/TEAD may recruit co-repressors, which could explain why XMU-MP-1 and YAP-5SA inhibited hormone-independent expression of *ESR1* and other ERα target genes. Consistent with our findings, a recent study showed that LATS1/2 maintained *ESR1* expression and ER^+^ breast cancer growth by inhibiting YAP/TAZ^40^. It would be interesting to determine why the binding of YAP/TEAD activates its canonical targets such as *CTGF* but represses other genes such as *ESR1*. It would be also interesting to determine whether TEAD can function as a cofactor for other oncogenic drivers that could be antagonized by YAP.

Breast cancer ranks number one in woman malignancy worldwide and is the second leading cause of cancer-related death among women^53^. ER^+^ breast cancer accounts for ~70% of breast cancer patients. Although anti-hormone therapy such as tamoxifen treatment can effectively control ER^+^ breast cancer progression, many treated patients will eventually develop resistance. Hence, it is urgent and necessary to develop alternative therapeutic strategies to treat ER^+^ breast cancer patients, especially those who have developed drug resistance. Our finding that YAP may inhibit ER^+^ breast cancer cell proliferation by blocking ERα transcriptional activity and *ESR1* expression open an exciting opportunity for developing novel therapeutics to treat ER^+^ breast cancer patients. Furthermore, our observation that MST1/2 inhibition could block the activity of commonly occurring hormone therapy-resistant ERα mutants suggests that targeting the Hippo pathway may provide a potential therapeutical approach to overcome endocrine therapy resistance conferred by ERα mutants. Although we have demonstrated that pharmacological inhibition of MST1/2 can impede ER^+^ breast cancer growth in vivo, the subcutaneous xenograft model employed in our study requires supraphysiological concentrations of estrogen and thus may compromise clinical relevance. A future study using more clinically relevant models such as intraductal xenograft^54^ may overcome this problem.

## Methods

### DNA constructs

The human ESR1/ERα full length and deletion constructs were described in a previous study^55^. The YAP-WT and YAP-5SA constructs were described previously^29^. YAP-5SAS94A was generated from YAP-5SA by PCR-based mutagenesis. The TEAD4 constructs were purchased from Origene (https://www.origene.com/; RC219686). TEAD4 full length and deletion constructs were amplified by PCR and the PCR products were subcloned into a *pcDNA3.1-FLAG* vector. To construct siTEAD4 insensitive TEAD4 (TEAD4^INS^), the primers were used: F: 5’- GGA GAA AGT TGA GAC AGA GTA TGC CAG ATA CGA GAA TGG-3’ and R: 5’- GTA TGC CAG ATA CGA GAA TGG ACA CTA CTC TTA CCG-3’. For the lentivirus infection, TEAD4^INS^ was amplified by PCR and subcloned into the *FUGW* vector and the YAP-WT or YAP-5SA were cloned into the *pLVX-IRES-ZSgreen* vector. To construct Tet-O-GFP, Tet-O-YAP5SA, Tet-O-YAP5SAS94A, and Tet-O-VGLL4, GFP, YAP5SA, YAP5SAS94A, and VGLL4 coding sequences were subclone into *pTet-O-Ngn2-puro* (Addgene plasmid, Cat. No. 52047*). pLenti CMV rtTA3 Hygro* (Addgene plasmid, Cat. No. 26730) was used to generate Dox-inducible cell lines. The ERα-WT (Cat. No. 49498), ERα-Y537S (Cat. No. 116374), and ERα-D538G (Cat. No. 49500) plasmids were acquired from Addgene (https://www.addgene.org/). The ERα constructs were amplified by PCR and the PCR products were subcloned into *pLVX-IRES-ZSgreen* vector.

### Cell culture

MCF-7, T47D, MDA-MB-231, and HEK293T cells are acquired form American Type Culture Collection (ATCC). T47D cells are maintained with RPMI-1640 (Gibco, Cat. No. 42401018) supplemented with 2 mM L-glutamine (Gibco, Cat. No. 25030081) and 10% FBS. HEK293T and MCF-7 cells are cultured with Dulbecco’s Modified Eagle’s Medium which contains 4.5 g/L glucose and 4 mM L-glutamine (DMEM, Gibco, Cat. No. 11965092) supplemented with 10% Fetal Bovine Serum (FBS, Gibco, Cat. No. 16000044). For hormone depletion conditions, MCF-7 cells are cultured with phenol red-free DMEM (DMEM, Gibco, Cat. No. 21063029) supplemented with 5% charcoal-stripped FBS (Gibco, Cat. No. A3382101) overnight before treatment. T47D cells are cultured with phenol red-free RPMI-1640 (RPMI-1640, Gibco, Cat. No. 11835030) supplemented with 5% charcoal-stripped FBS (Gibco, Cat. No. A3382101) overnight before treatment. All cell lines are characterized by cell line authentication via Short Tandem Repeat (STR) is performed via PowerPlex 21 system. The STR data of MCF-7 and T47D cell lines are found consistent with STR data in ATCC. For packaging lentivirus, the HEK293T cells were transfected with the expression vectors and package vectors (psPAX2 and pMD2.G) by PolyJet (SignaGen laboratories, Cat. No. SL100688). After 48 h, the viruses were harvested for cell line infection with the standard method.

### Reagents

The reagents used were: XMU-MP-1 (MCE, Cat. No. HY-100526), Doxycycline (Dox) (Sigma, Cat. No. 33429), 17β-estradiol (E2) (Sigma, Cat. No. E8875), Hygromycin B (Sigma, Cat. No. 10843555001), Puromycin (Sigma, Cat. No. P9620), MG132 (Calbiochem, Cat. No. 474790), Leptomycin B (LMB) (Santa Cruz, Cat. No. sc-358688), and Fulvestrant (Beyotime Company, Cat. No. SD0002).

### Immunoblot analysis

Cells were harvested and lysed with RIPA buffer. Proteins were separated by electrophoresis on SDS-polyacrylamide gel electrophoresis (PAGE) and electro-transferred to PVDF membrane. Membranes were washed with TBST and incubated with primary antibodies for 2 h. And then the membranes were washed three times with TBST and incubated with second antibodies for 2 h, after being washed three times with TBST, the membranes were probed with ECL system (Cytiva, Cat. No. RPN2105). The antibodies used in this study were listed here: Anti- YAP (Santa Cruz, Cat. No. sc-101199); Anti- ERα (Cell Signaling Technology, Cat. No. sc-D8H8); Anti-TEAD4 (Santa Cruz, Cat. No. sc-390578); Anti-TEAD1(BD Transduction Laboratories, Cat. No. 610922); Anti-HA (COVANCE, Cat. No. MMS-101R); Anti-Myc (Abcam, Cat. No. ab32); Anti-Myc (Abcam, Cat. No. Ab9106); Anti-Flag (Abcam, Cat. No. Ab49763,), Peroxidase-Conjugated AffiniPure Goat Anti-Mouse IgG (Jackson ImmunoResearch Code,115-035-003), or Goat Anti-Rabbit IgG (Jackson ImmunoResearch, Code,111-035-144). Chemiluminescent signals were visualized with ECL system (Cytiva, Cat. No. RPN2105).

### Co-immunoprecipitation assay

Immunoprecipitation was performed according to standard protocol. MCF-7 cell lysates were incubated with protein-A–Sepharose beads (Thermo scientific, Cat. No 53139) for 1 h at 4 °C to eliminate non-specific binding proteins. After removal of the protein-A beads by centrifugation, the cleared lysates were incubated with antibodies for ERα (Santa Cruz, Cat. No. SC8005,), YAP (Santa Cruz, Cat. No. sc-101199), or TEAD4 (Santa Cruz, Cat. No. sc-390578) for an overnight while mouse IgG was used as the negative control. After incubation, it was immobilized and precipitated with Protein-A resin. The bounded proteins were analyzed by western blot. For overexpression experiments, HEK293T cells were transfected with 5 μg HA-ERα (full length or deletion mutants) and Flag-TEAD4 plasmids (full length or deletion mutants) or full-length GFP-TEAD4 in the absence or presence of Flag-YAP in 10 cm dishes. Cell lysates were precleared with protein-A–Sepharose beads and subsequently incubated with HA (COVANCE, Cat. No. MMS-101R) or GFP (Santa Cruz, Cat. No. sc-9996) antibody overnight. After incubation, it was immobilized and precipitated with Protein-A resin. The bound proteins were analyzed by immunoblot assay.

### Immunofluorescence assay

MCF-7 cells were fixed with 4% paraformaldehyde in PBS for 10 min, permeabilized with 0.2% Triton X-100 (Sigma, Cat. No. T8787) for 5 min, and blocked by 5% BSA in PBS for 1 h. A rabbit anti-ERα polyclonal antibody (Cell Signaling Technology, Cat. No. 8644 S) and mouse anti-YAP monoclonal antibodies (Santa Cruz, Cat. No. sc-101199) were used, followed by Alexa Flour 647 anti-rabbit antibody (Invitrogen, Cat. No. A-31573) and FITC-conjugated anti-mouse antibodies (Jackson ImmunoResearch, 715095150). As negative controls, the samples were incubated with the secondary antibodies without primary antibodies. Images were acquired under conditions fulfilling the Nyquist criterion using Nikon A + laser scanning confocal system with a ×60 oil NA1.4 objective and pinhole size of 1.0 Airy Unit. The acquired pictures were further processed and assembled using ImageJ.

### RNA interference

For RNAi experiments in breast cancer cells, the siRNAs were acquired from Sigma–Aldrich. The sequences for YAP silencing were: 5’- CAC CUA UCA CUC UCG AGA U-3’ and 5’-GCU CAU UCC UCU CCA GCU U-3’. The sequences for negative control were: 5’-UUC UCC GAA CGU GUC ACG U-3’. The sequences for TEAD4 silencing were: 5’- CUC GCU AUG AGA AUG GAC A −3’ and 5’- CAG AGU AUG CUC GCU AUG A −3’. The sequences for TEAD1 silencing were: 5’- CUC CUU UGG GAA GCA AGU A −3’ and 5’- CAU UCA GGU UCU UGC CAG A −3’. The sequences for TEAD2 silencing were: 5’- GUG GUG AAU UUC UUG CAC A-3’ and 5’- GAU GUA UGG UCG GAA UGA A-3’. The sequences for TEAD1/3/4 silencing were: 5’- AUG AUC AAC UUCA UCC ACA AG −3’ and 5’ GAU CAA CUU CAU CCA CAA GCU −3’. The RNAi MAX reagent (Invitrogen Cat. No. 13778150) was used for the transfection of siRNA. The knockdown efficiency was validated via real-time PCR and immunoblotting.

### Virus infection and transient transfection

For the virus infection, the expression vectors were transfected together with package vectors (*psPAX2* and *pMD2.G*) into HEK293T cells by Lipofectamine 2000 (Thermo Fisher Scientific, Cat. No. 11668) or by PolyJet (SignaGen laboratories, Cat. No. SL100688). After 48 h, the supernatants of the medium were collected and filtered with 0.45 μm filter. The supernatant containing the virus was stored at 4 °C for cell infection. The breast cancer cells were cultured in fresh media and subsequently infected with lentivirus overnight together with Polybrene (Sigma, Cat. No. H9268). For cell transfection, Lipofectamine 2000 (Invitrogen) or PolyJet was utilized according to the protocol.

### RNA extraction and RT-qPCR analysis

We extracted the total RNA by RNeasy plus mini kits according to the protocol (Qiagen, Cat. No. 74106). After RNA extraction, the RNA was subjected to reverse transcription PCR for cDNA synthesis according to the RT-PCR kit (Applied Biosystems, Cat. No. 4368814). The relative gene expression was measured according to 2^−ΔΔCT^ methods. The housekeeping gene 36B4 was used for internal control. The Primer sequences were: YAP, F: TCC ACC AGT GCA GCA GAA TA, R: TTG GGT CTA GCC AAG AGG TG. CTGF, F: CTC GCG GCT TAC CGA CTG, R: GGC TCT GCT TCT CTA GCC TG. CYR61, F: AGC AGC CTG AAA AAG GGC AA, R: AGC CTG TAG AAG GGA AAC GC. 36B4, F: GGC GAC CTG GAA GTC CAA CT; R: CCA TCA GCA CCA CAG CCT TC. GREB1, F: CGT GTG GTG ACT GGA GTA GC, R: ACC TCT TCA AAG CGT GTC GT. ESR1, F: GCT ACG AAG TGG GAA TGA AAG, R: TCT GGC GCT TGT GTT TCA AC. PS2 (TFF1), F: CAT CGA CGT CCC TCC AGA AGA G, R: CTC TGG GAC TAA TCA CCG TGC TG. CCND1, F: ATC AAG TGT GAC CCG GAC TG, R: CTT GGG GTC CAT GTT CTG CT. PDZK1, F: CCT GAG TGA ACG AAC AGA GC, R: TCT CTG CTG GGC TACACTTC.

### ChIP-qPCR

ChIP (Chromatin Immunoprecipitation) assay was performed as previously study description^56^, In brief, cells were crosslinked by adding formaldehyde to a final concentration of 1% for 10 min only or 2 mM DSG crosslinker (CovaChem, Cat. No.13301) at room temperature for 1 h followed by secondary fixation with 1% formaldehyde (Pierce, Cat. No. 28908) for 10 min and quenched by adding glycine. Subsequently, cells were washed with PBS and subject to cell lysis. The cell extracts were subject to sonication. After centrifuge, the cell extracts were incubated with prepared ERα/TEAD/YAP antibody-Dynabeads (Invitrogen, Cat. No. 11031) together, washed with buffers, and de-crosslinked at 65 °C overnight. The antibodies used in ChIP-qPCR were anti-ERα (Santa Cruz, Cat. No. sc-8002x), anti-TEAD4 (Santa Cruz, Cat. No. sc-101184), and anti-YAP (Santa Cruz, Cat. No. sc-271134). The enriched DNA was extracted via DNA extraction kits (Qiagen, Cat. No. 28106) and subject to quantitative PCR analysis. The Primer sequences for ChIP-qPCR were demonstrated here: ESR1, F: AAG CAA GGG AGG AAT GCC AG, R: AGG CAT AGC TCA CTC CTG TC; GREB1, F: GGC TCC AGT CCA AGT ACA CA, R: GCC CTG AAG TGT TTT GC TGG; TFF1 (PS2), F: GGC AGG CTC TGT TTG CTT AAA, R: TTC CAT GTA GCT TGA CCA TGT CT; PDZK1, F: AGG CCC AGC AAA GAC AAA TG, R: AAA CCA CAG GCT GAG GAC TG; CCND1, F: AAC AAA ACC AAT TAG GAA CCT T, R: CTT GGG GTC CAT GTT CTG CT; SLC9A3R1, F: GAG GCA GAT GGC AGT CAG AAT, R: CAG TGG ATA GTC AAG GCT CCC; CA12, F: CTC AGT TTC AGG CGG GAT CA, R: CAG AAA CAC GGC AAG GGA CT; COX6C, F: ATC TTT AGC TTC CAC ATG CTC TTC T, R: AATGTGGCAGTGTCAGGGTGA; CTGF, F:TGT GCC AGC TTT TTC AGA CG, R:TGA GCT GAA TGG AGT CCT ACA CA.

### RNA sequencing and data analysis

The global gene expression analysis (Vehicle vs XMU-MP-1 treated groups) was based on RNA sequencing platform from BGI (Beijing Genomic Institute). Cellular RNA was extracted using Qiagen RNA extraction kit (Qiagen; Cat: 74104) according to the manufacturer’s instructions. The cellular RNA was sent to BGI Genomics (https://www.bgi.com) for RNA sequencing. RNA was quality-accessed with an Agilent 2100 Bioanalyzer (Agilent RNA 6000 Nano Kit) with RNA integrity number above 9 for library construction. The total RNA was used for library construction according to the protocol of BGISEQ-500 platform. The libraries were sequenced using BGISEQ-500 platform. Then the FASTQ sequencing files were aligned to the hg19 human genome using STAR aligner with uniquely mapped reads kept for further analysis. Differential expression was analyzed using DESeq2 with default parameters. The RNA sequence data are deposited in the Gene Expression Omnibus (GEO) database (Assessing number: GSE165288). Analysis was performed for differentially expressed genes (*P* < 0.01 and fold change > 2) by Ingenuity Pathway Analysis (IPA). For gene set enrichment analysis of RNA-seq data, gene sets of Hallmark Estrogen Response Early and Cordenonsi YAP Conserved Signature were used and downloaded from Molecular Signatures Database v7.4, GSEA was implemented using the GSEA 4.1.0 software, with default parameters. RNA-seq data of YAP-5SA overexpressing MCF-7 were retrieved from GEO (https://www.ncbi.nlm.nih.gov/geo/) under accession GSE107010. The volcano plot was generated using ‘ggplot2’ package in R (threshold *P* < 0.05 and fold change > 1.5).

### ChIP-seq experiment and data analysis

For XMU-MP-1 treatment experiments, ChIP-Seq libraries were generated using KAPA HTP Library Preparation Kit Illumina® platforms (KAPA, KR0426) according to the manufacturer’s protocol. Briefly, the ChIP DNA is quantified on the Qubit® 2.0 Fluorometer, after ChIP DNA end-repaired and 3’ ends adenylated, barcoded with mµltiplex adapters were ligated to ChIP DNA. PCR amplified libraries are purified and size selected with Ampure XP beads, then checked on the Agilent 2100 Bioanalyzer to ensure the library is the expected size. After samples were quantified, normalized, and pooled, final samples run on the Illumina HiSeq 2500 using the 75 bp high output sequencing kit for single-end sequencing at UTSW next-generation sequencing core. Reads were trimmed and aligned to the human genome (hg19) using ‘Bowtie2’, reads were sorted using “Samtools” subsequently. The ChIP-seq data are deposited in the Gene Expression Omnibus (GEO) database (Assessing number: GSE197239). ChIP-seq data for ERα, TEAD, and YAP co-binding ChIP-seq analysis shown in Supplementary Fig. 6 were retrieved from GEO under accession GSE72249 and GSE107013. SRA files were downloaded for re-analysis. Peaks were identified using MACS2 v2.1.1 with the *p*-value cutoff 1e-5. After peak calling, ENCODE human blacklisted regions were removed from the peak files subsequently. ChIP-seq signal tracks were generated by ‘DeepTools’ and normalized by RPKM. Signal tracks were visualized by Integrative Genomics Viewer (IGV) software. Peak overlapping analysis and subsets annotation were performed using ‘HOMER’ v4.9. Heatmaps and signal plots for the ChIP-seq peak subsets were generated using ‘DeepTools’.

### Relapse-free survival data analysis

The relapse-free survival (RFS) survival data of YAP was generated from KMPLOT online analysis database (http://kmplot.com/analysis/index.php?p=service&cancer=breast). The gene affy ID is 224894_at. “Auto selected best cutoff” and “ER-positive” were selected for ER-positive breast cancer analysis. “Auto selected best cutoff” and “ER-negative” were selected for ER-negative breast cancer analysis. The RFS survival data of *TEAD4* in endocrine-treated patients was generated from KMPLOT database. The gene affy ID is 41037_at. “Auto selected best cutoff” and “patients with endocrine therapy” were selected for analysis. The RFS survival data of *MST1* (*STK4*), *MST2* (*STK3*), and *TAZ* (*WWTR1*) in endocrine-treated patients were generated from KMPLOT database. The gene affy IDs are 225364 (*MST1*), 204068 (*MST2*), and 202133(*TAZ*)_at. “Auto selected best cutoff” and “patients with endocrine therapy” were selected for analysis. The RFS survival of YAP in the different databases was generated from KMPLOT database. The gene affy ID is 224894_at. “Auto selected best cutoff” and “ER-positive” were selected for ER-positive breast cancer analysis. “GSE16931”, “GSE9195”, “E-MTAB-365”, and “GSE21653” were used for different datasets.

### Analysis of TCGA breast cancer datasets

Gene expression data for 1218 TCGA breast cancer patients were downloaded from the webpage (http://xena.ucsc.edu/), the ER high expression group is the value of ESR1 Log2(FPKM-UQ + 1) ≥9.49. MORPHEUS (https://software.broadinstitute.org/morpheus/) was used for the generation of the gene expression heatmap (data normalization was performed by z-score) and the gene correlation heatmap (Spearman correlation analysis among genes was performed by Graphpad Prism 8). YAP tumor RNA-seq (TCGA) in Breast cancer can be downloaded from Genomic Data Commons (GDC) data portal website (https://portal.gdc.cancer.gov/). YAP expression analysis in ER-positive and ER-negative breast cancer tissues and normal tissues were performed by Graphpad Prism 8.

### Analysis of METABRIC breast cancer datasets

METABRIC breast cancer data were downloaded from cbioportal (https://www.cbioportal.org/). KMPLOT online analysis platform (http://kmplot.com/analysis/index.php?p=service&cancer=custom_plot) was used for the generation of Kaplan–Meier graph of recurrence-free survival for YAP in ERα-positive and ERα-negative breast cancer in METABRIC cohort. Prognostic analysis of the 7-gene YAP/TAZ signature (*YAP1*, *TAZ*, *CTGF*, *CYR61*, *AMOTL2*, *DAB2*, *DKK1*) model was performed using the sangerbox tools (http://vip.sangerbox.com/), a free online platform for data analysis and visualization. Briefly, the prognosis risk score is the sum of products of the expression level of each gene and its corresponding regression coefficient, the risk score was established with the following formula: score $={\sum }_{i=1}^{n}{{{{{\rm{\beta }}}}}}i{Exp}i$. The patients were stratified into high-risk and low-risk groups based on the optimal-cutoff value of the risk score. For Kaplan–Meier curves, p-values and hazard ratio (HR) with 95% confidence interval (CI) were generated by log-rank tests and univariate Cox proportional hazards regression.

### Cell proliferation assay

MCF-7, T47D, and MDA-MB-231 cells were transfected with siYAP, siTEAD4, or scramble siRNAs in 24-well plates. Twenty four hours after transfection, the cell number was counted, and 4000 cells were seeded into 96-well plates. The relative cell viability was measured at indicated time points. For XMU-MP-1 or tamoxifen (Sigma Cat. No. T5648) assays for cell proliferation, the cells were treated with indicated drug concentrations. The relative cell viability was measured at indicated time points. Cell numbers were determined using the WST-1 cell proliferation reagent (Sigma–Aldrich, Cat. No. 5015944001).

### Xenograft and PDX tumor models

The procedures for all animal experiments were reviewed and approved by the IACUC of UT Southwestern Medical School. All mice were kept under a specific pathogen-free (SPF) and temperature-controlled environment. Housing condition for mice: 20 ± 2 °C, 50 ± 5% humidity, 12–12 h light–dark cycles. Female 6-week-old NOD scid gamma (NSG, NOD.Cg-Prkdc(scid)Il2rg(tm1Wji)/SzJ strain, The Jackson Laboratory, Maine, USA) mice were estrogen-supplemented by s.c. implantation of slow-release 17β-estradiol pellets (0.72 mg/90-d release; Innovative Research of America) 1 day before tumor cell injection into the mammary fat pad (2 × 10^6^ MCF-7 cells suspended in a 100 ul Matrigel solution). When tumor xenografts reached a mean volume of ∼60 mm^3^ (length × width^2^ /2), mice were randomly assigned to experimental treatment groups (7–8 mice/ group). XMU-MP-1 (dissolved in 0.1% citric acid aqueous solution containing 20% Kolliphor HS 15.) was administered intraperitoneally daily with 3 mg or 10 mg/kg for up to one month, and the control group was injected with the solvent. Dox in PBS was injected intraperitoneally with 20 mg/kg every day. Patient-derived breast cancer cells WHIM20 (Horizon Discovery, St. Louis, MO) was derived from skin metastasis of breast cancer patients and characterized as HER2^−^, ER^+^, and PR^+^. WHIM20 tumor cells (5 × 10^5^ cells) suspended in 100 ul Matrigel and DMEM mixture (1:1) were subcutaneously injected into NSG mice (without any treatment). Tumor size was measured every week after visible and XMU-MP-1 treatment was the same as indicated above.

### Patient-derived explant (PDEx) assay

The discarded surgical samples for research purposes were approved by the ethnical committee of Qilu Hospital, Shandong University. The excised tissues were processed according to the ex vivo culture protocol. In general, breast cancer tissues were cultured on gelatin sponges with the cell culture medium containing 10% FBS. The tissues were either treated with vehicle or XMU-MP-1 for 48 h. Subsequently, tissues were fixed in 10% formaldehyde at 4 °C overnight. The tissues were stained with hematoxylin and eosin to confirm the quality. After that, immunohistochemistry was performed to examine the indicated markers.

### Clinical breast tumor samples

One hundred and forty paraffin-embedded breast cancer samples were collected from the Department of Pathology, Shandong Qilu Hospital. All the breast tumor samples were examined by ERα status, PR status, HER2 status by pathological specialists. The pathological grade plus lymph node metastasis status of each sample was also examined by pathological specialists. This study was reviewed and approved by the Ethical Board at the Qilu Hospital of Shandong University with written informed consent from all the patients. A specific antibody against YAP (cat: 101199; Santa Cruz) was used to detect the staining density in human samples. The scores were calculated on the intensity and percentage of positive tumor cells in the whole tissue, which were evaluated according to the Fromowitz Standard. The staining intensity was graded as: no staining, 0; weakly positive, 1; moderately positive, 2 and strongly positive, 3. The percentage for positive cells was into four grades: 0–25% staining, 1; 26–50% staining, 2; 51–75% staining, 3 and 76–100% staining, 4. All staining was assessed at ×200 magnifications and at least three fields from each core were counted.

### Statistics and reproducibility

ChIP-seq and xenograft experiments are based on one set of experiments. All other experiments were performed at least three independent times unless noted otherwise. Two-side unpaired Student’s *t*-test was used for comparisons. A *P*-value of <0.05 is considered to be significant. Error bars on the graphs were presented as the s.d.

### Reporting summary

Further information on research design is available in the Nature Research Reporting Summary linked to this article.

## Supplementary information

Supplementary Information

Description of Additional Supplementary Files

Supplementary Data 1

Supplementary Data 2

Supplementary Data 3

Reporting Summary

## Data Availability

The data of Fig. 1c–e and Supplementary Figs. 1a–d are available at TCGA website. The Data of Supplementary Fig. 1i and j are available at METABRIC database website. The RNA-seq data are available in the GEO database with accession number GSE165288 and GSE107010, the ChIP-seq data are available in the GEO database with accession number GSE197239, GSE72249, and GSE107013. All the other data are available within the article and its Supplementary Information. Source data are provided with this paper.

## Source data

Source Data
