# Supplementary material for: YAP inhibits ERα and ER+ breast cancer growth by disrupting a TEAD-ERα signaling axis
Source: Nat Commun. 2022 Jun 2;13:3075. doi: 10.1038/s41467-022-30831-5 (PMC9163075; doi:10.1038/s41467-022-30831-5)
Supplement: Supplementary file 1 — Supplementary Information [file 41467_2022_30831_MOESM1_ESM.pdf]

Supplementary information for:

YAP inhibits ERα and ER+ breast cancer growth by disrupting a TEAD-ERα signaling axis

Xu Li[1, #], Shu Zhuo[1,2,12,#], Ting Zhuang[3,#], Yong Suk Cho[1], Guojin Wu[4], Yuchen Liu[2,5,6], Kun Mu[7, 8], Kai Zhang[9], Peng Su[7], Yingzi Yang[2, 5, 6], Cheng Cheng Zhang[4], Jian Zhu[1, 3,10, #, *], Jin Jiang[1,11*]

[1]Department of Molecular Biology, University of Texas Southwestern Medical Center, Dallas, TX 75390, USA
[2]Department of Developmental Biology, Harvard School of Dental Medicine, 188 Longwood Ave. Boston, MA 02215, USA
[3]Henan Key Laboratory of Immunology and Targeted Drugs, School of Laboratory Medicine, Xinxiang Medical University, 453000, P.R China
[4]Department of Physiology, University of Texas Southwestern Medical Center, Dallas, TX 75390, USA
[5]Harvard Stem Cell Institute, 188 Longwood Ave. Boston, MA 02215, USA
[6]Dana-Farber/Harvard Cancer Center, 188 Longwood Ave. Boston, MA 02215, USA
[7]Department of Pathology, Qilu Hospital, Cheeloo College of Medicine, Shandong University, Jinan, Shandong 250012, P.R China
[8]Department of Pathology, School of Basic Medical Sciences, Cheeloo College of Medicine, Shandong University, Jinan, Shandong 250012, P.R China
[9]Department of Breast Surgery, Qilu Hospital, Cheeloo College of Medicine, Shandong University, Jinan, Shandong 250012, P.R China
[10]Department of General Surgery, the Second Hospital, Cheeloo College of Medicine, Shandong University, Jinan, Shandong 250012, P.R China
[11]Department of Pharmacology, University of Texas Southwestern Medical Center, Dallas, TX 75390, USA
[12]Current address: Signet Therapeutics Inc., Research Institute of Tsinghua University In Shenzhen, Shenzhen, Guangdong 518057, P.R. China

[#]These authors contribute equally

*Corresponding authors: jian.zhu@email.sdu.edu.cn, jin.jiang@utsouthwestern.edu

Supplementary Figure 1-11.

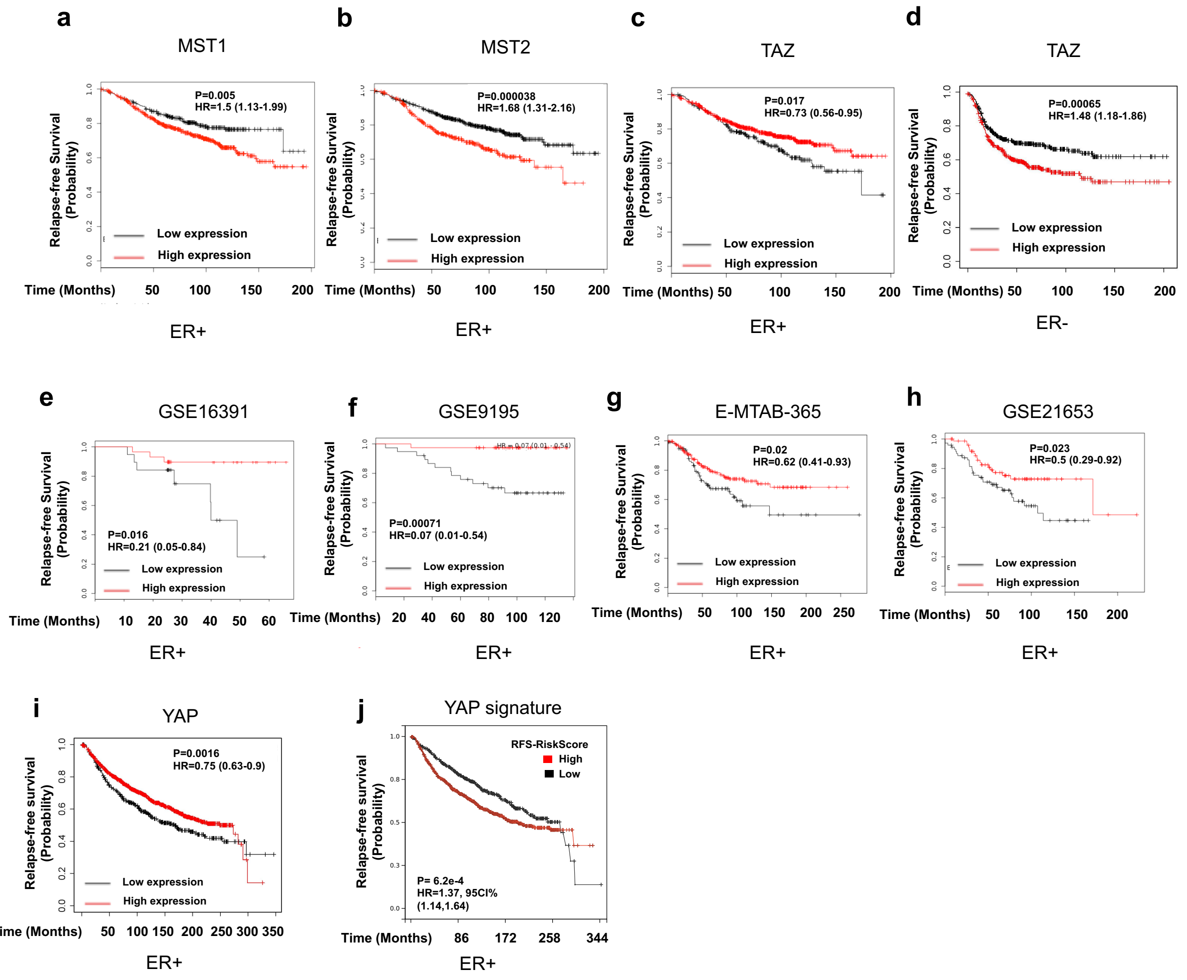

Supplementary Fig. 1 Kaplan–Meier analysis of relapse free survival for MST1/2 and Yap/TAZ

a, Relapse free survival analysis with low versus high expression of *MST1* in ER$^+$ breast cancer patients from TCGA/BRCA.
b, Relapse free survival analysis with low versus high expression of *MST2* in ER$^+$ breast cancer patients from TCGA/BRCA.
c,d, Relapse free survival analysis with low versus high expression of *TAZ* in ER$^+$ (**c**) or ER$^-$ (**d**) breast cancer patients from TCGA/BRCA.
e-h, Relapse free survival analysis with low versus high expression of *YAP* in ER$^+$ breast cancer patients from GSE16391, GSE9195, E-MTAB-365, and GSE21653 Datasets.
i, Relapse free survival analysis with low versus high expression of *YAP* in ER$^+$ breast cancer patients from the METABRIC cohort.
j, YAP signature (*YAP1*, *TAZ*, *CTGF*, *CYR61*, *AMOTL2*, *DAB2*, *DKK1*) is associated with low risk in ER$^+$ breast cancer patients from the METABRIC cohort.
Two-sided log-rank test were used for **a**-**j**.

Fig. S2

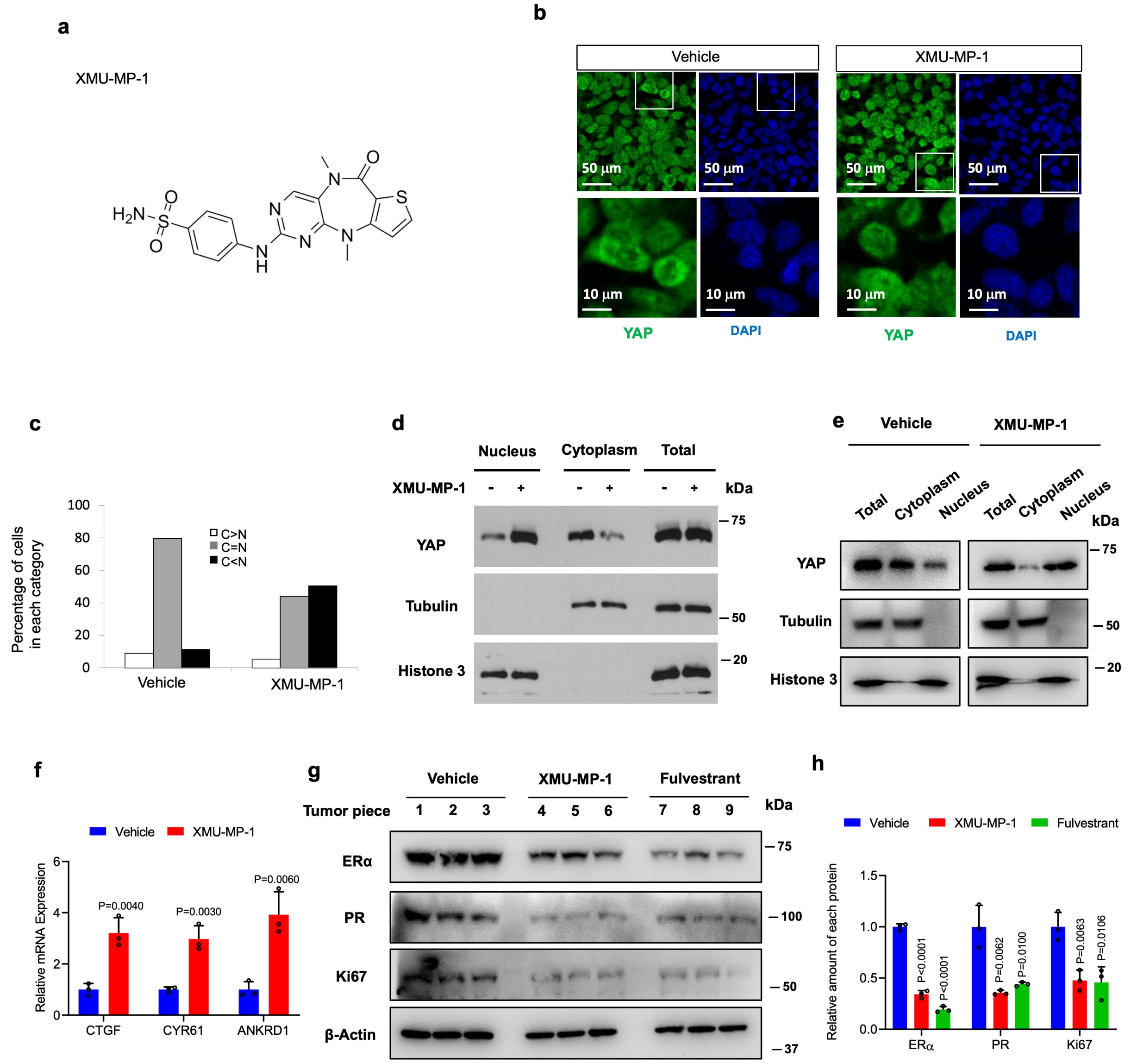

Supplementary Fig. 2 Evaluation of the effect of XMU-MP-1 on ER$^+$ breast cancer

a, Chemical structure of the MST1/2 inhibitor XMU-MP-1.
b, Immunofluorescence staining of YAP in MCF-7 after treatment with either vehicle or 2uM XMU-MP-1 for 12 h. Enlarged images of the outlined regions are shown at bottom.
c, Quantification of cells with the indicated relative levels of cytoplasmic (C) and nuclear (N) YAP from the experiments described in (**b**). Results are representative of two independent experiments.
d, Western blot analysis to detect YAP in the nuclear (N) and cytoplasmic (C) fraction or whole cell lysates (WC) in MCF-7 treated with either vehicle or 2μM XMU-MP-1. Tubulin and Histone3 were used for cytoplasm and nuclear marker, respectively. Results are representative of two independent experiments.

e, f, XMU-MP-1 promoted the nuclear translocation of YAP and activity in xenografts. Female NOD scid gamma (NSG) mice bearing MCF-7 tumors were treated daily with vehicle or 10mg/kg XMU-MP-1. After a week, the tumors were fixed and lysed, followed by western blot analysis to detect YAP in the nuclear or cytoplasmic fraction, or total cell lysates (**e**). RT-qPCR was performed to analyze the expression of the indicated YAP target genes (**f**). Results are representative of three independent experiments.

g, h, ER^+ breast cancer sample from the same patient was cut into 9 pieces and cultured on gelatin sponges, treated with vehicle, 3 μM XMU-MP-1, or 0.2 μM Fulvestrant for 48 hours. Tumor samples were lysed for western blot analysis with the indicated antibodies (**g**), n=3. The expression levels of ER, PR and Ki67 were quantified (**h**), results are representatives of 3 independent experiments.

Data are means ± s.d. Two-sided, unpaired t-test were used for **f**, **h**. Source data are provided in the Source Data file.

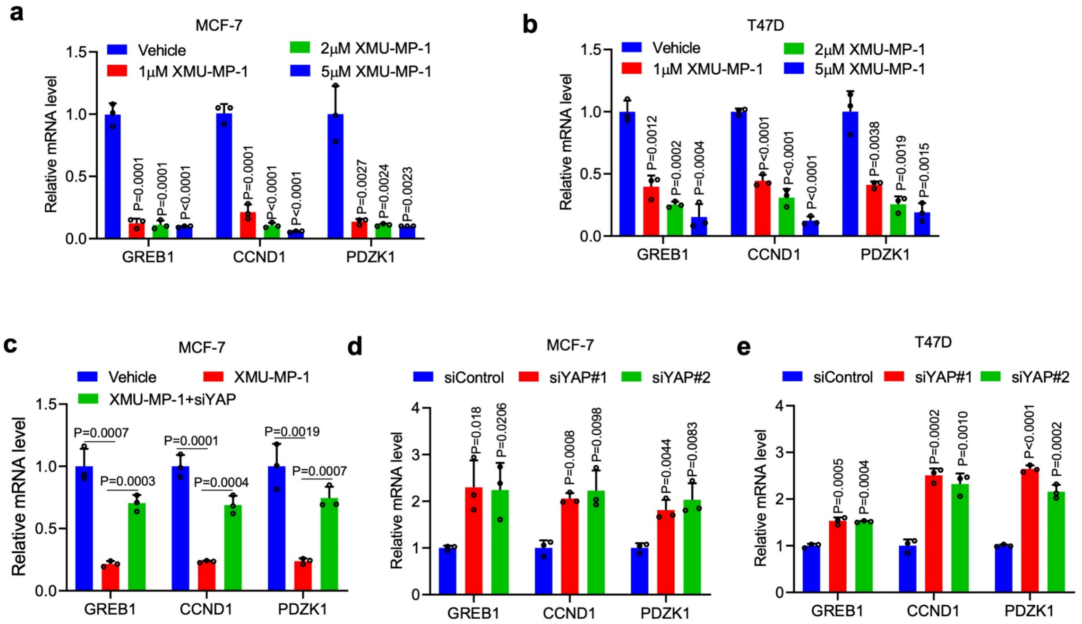

Supplementary Fig. 3 XMU-MP-1 inhibits ERα signaling in ER positive breast cancer cells

a,b, MCF-7 or T47D cells were treated with either vehicle or XMU-MP-1with indicated concentrations for 12 h, followed by RT-qPCR analysis of mRNA expression of *GREB1*, *CCND1*, and *PDZK1*.
c, MCF-7 cells were treated with either Control or YAP siRNA for 24h and then with either vehicle or 2μM XMU-MP-1 for 12 h, followed by RT-qPCR analysis of mRNA expression of the indicated ERα target genes.
d,e, MCF-7 or T47D cells were treated with either Control or YAP siRNA for 48h, followed by RT-qPCR analysis of mRNA expression of the indicated ERα target genes. .
Results shown in **a-e** are representative of 3 independent experiments. Data are means ± s.d. Two-sided, unpaired t-test were used for **a-e**. Source data are provided in the Source Data file.

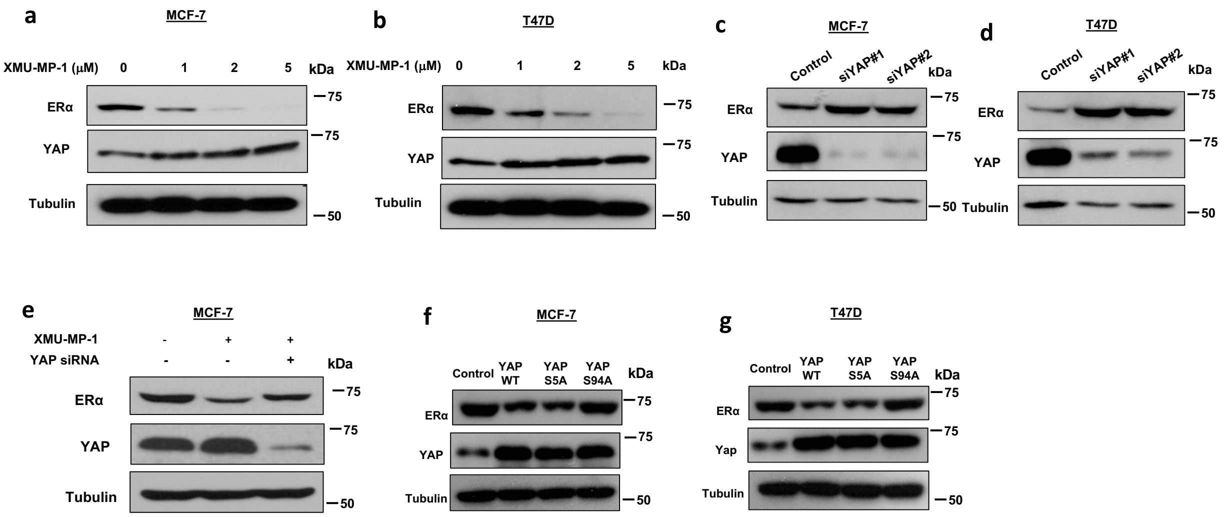

Supplementary Fig. 4 XMU-MP-1decreased ERα protein level in ER positive breast cancer cells

a,b, Western blot analysis of ERα and YAP expression in MCF-7 or T47D cells treated with either vehicle or XMU-MP-1 with the indicated concentrations for 12 h.
c,d, Western blot analysis of ERα and YAP expression in MCF-7 and T47D cells treated with either Control or YAP siRNA for 48h.
e, Western blot analysis of ERα and YAP expression in MCF-7 cells treated with Control or YAP siRNA for 24h, followed by treatment with vehicle or 2 μM XMU-MP-1 for another 12 h.
f,g, Western blot analysis of ERα and YAP expression in MCF-7 and T47D cells transfected with the indicated lentiviral constructs for 48h.
Results in **a-g** are representative of 3 independent experiments.

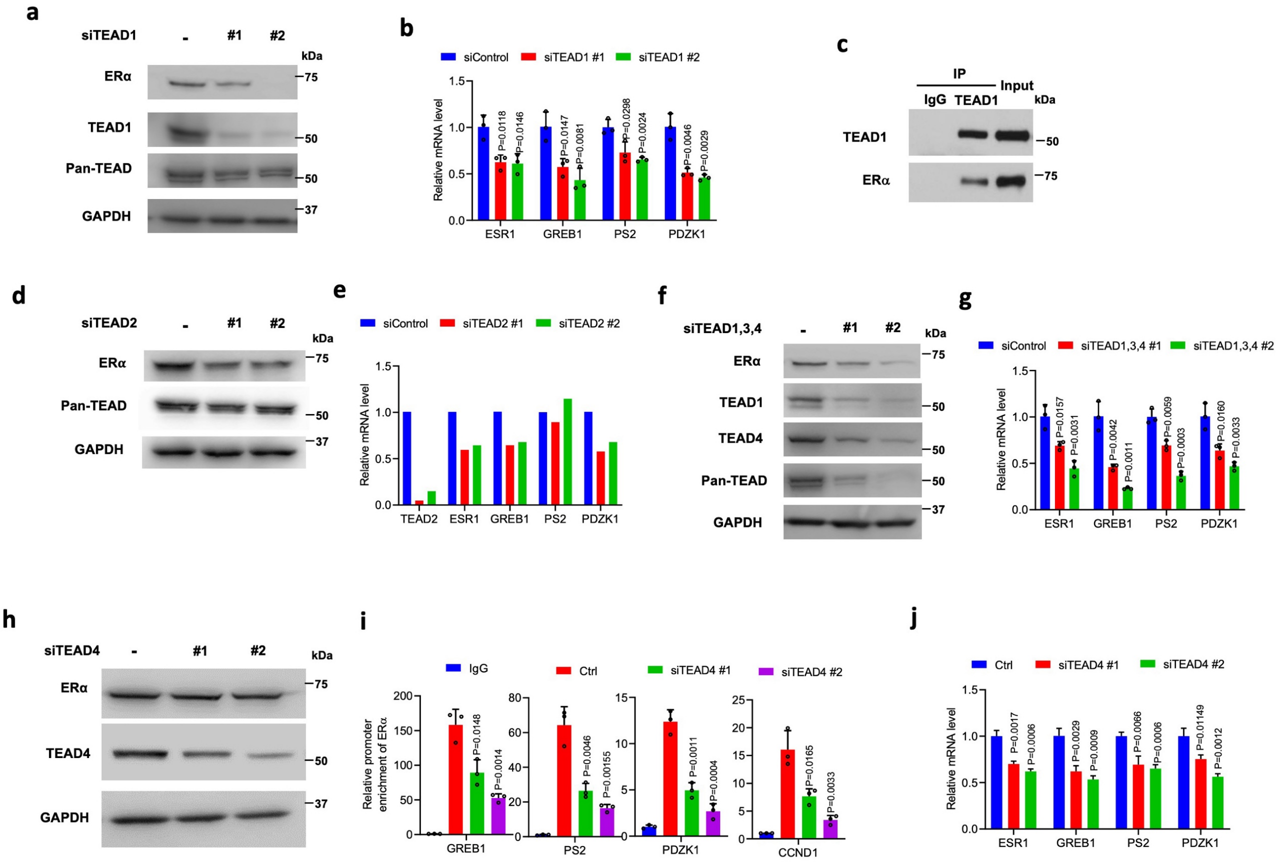

Supplementary Fig. 5 TEAD regulates ERα transcriptional activity

a,b, TEAD1 depletion reduced ERα target gene expression. MCF-7 cells transfected with control (Ctrl) or two independent TEAD1 siRNAs were grown for 48 hours, followed by western blot analysis of ERα, TEAD1, and pan-TEAD expression (**a**) or RT-qPCR analysis of the indicated ERα target genes (**b**).
c, Co-IP experiments revealed that ERα formed a complex with TEAD1.
d,e, TEAD2 depletion reduced ERα target gene expression. MCF-7 cells transfected with control (Ctrl) or two independent TEAD2 siRNAs were grown for 48 hours, followed by western blot analysis of ERα and pan-TEAD expression (**d**) or RT-qPCR analysis of the indicated ERα target genes (**e**). Data presented are from one set experiments.
f,g, Depletion of multiple TEAD family members reduced ERα target gene expression. MCF-7 cells transfected with control (Ctrl) or two independent TEAD1, 3, 4 siRNAs were grown for 48 hours, followed by western blot analysis of ERα, , TEAD1, TEAD4 and pan-TEAD expression (**f**) or RT-qPCR analysis of the indicated ERα target genes (**g**).
h-j, MCF-7 cells grown in hormone depletion medium were treated with either control or TEAD4 siRNA for 45 hrs. The cells were then treated with 10 nM E2 for 3 hrs., followed by western blot analysis for the expression of ERα and TEAD4 (**h**), ChIP qPCR (**i**) to determine the binding of ERα to the promoter/enhancer regions of its target genes, RT-qPCR analysis to monitor the expression of the indicated ERα target genes (**j**).
Results shown in **a-c, f-j** are representative of 3 independent experiments. Data are means ± s.d. Two-sided, unpaired t-test were used for **b**, **g**, **i-j**. Source data are provided in the Source Data file.

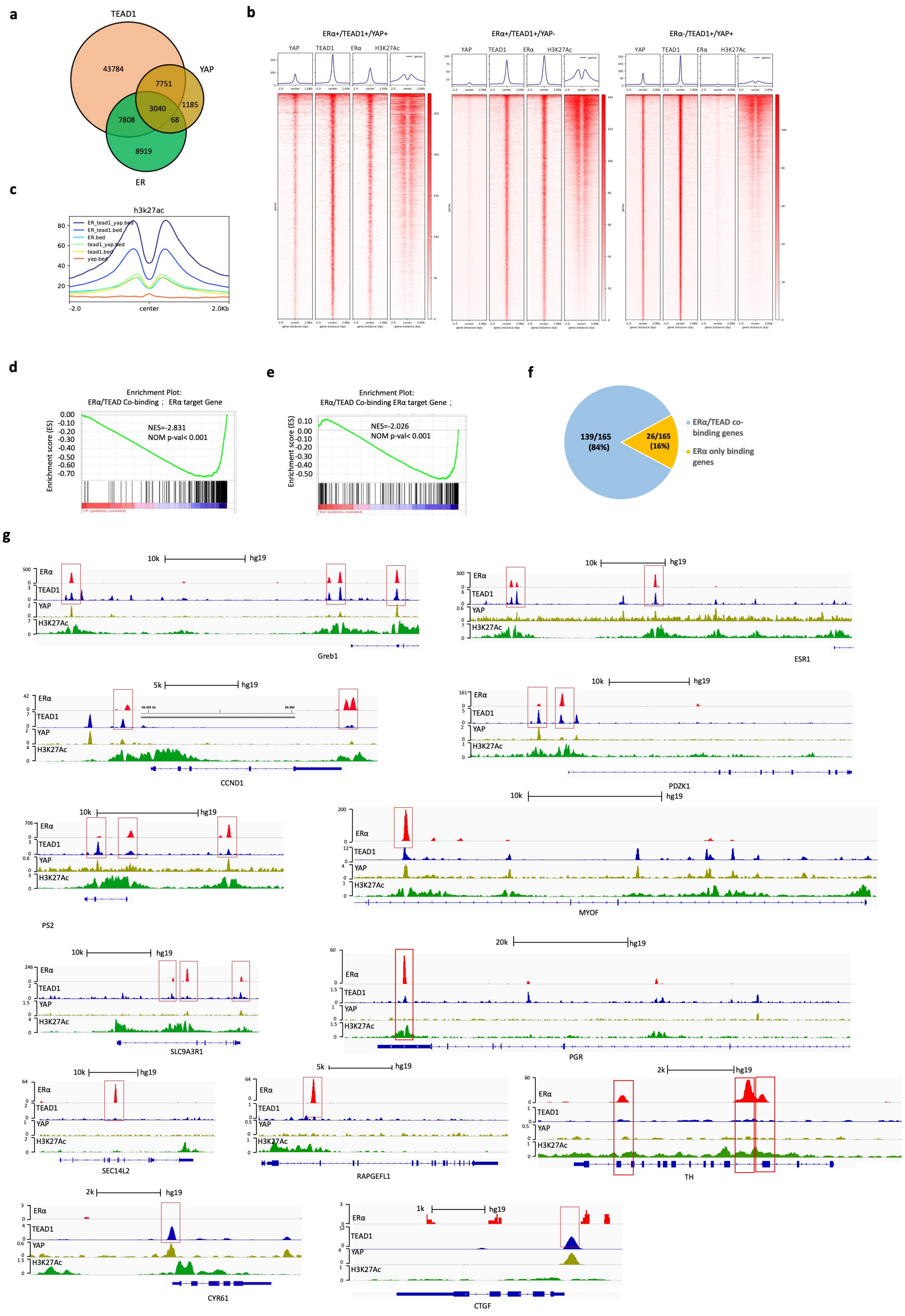

a
TEADI
YAP
ER
43784
7751
3040
1185
7808
68
8919
b
ERα+/TEAD1+/YAP+
ERα+/TEAD1+/YAP-
ERα-/TEAD1+/YAP+
YAP
TEAD1
ERα
H3K27Ac
c
h3k27ac
ER_tead1_yap.bed
ER_tead1.bed
ER.bed
tead1_yap.bed
tead1.bed
yap.bed
-2.0
center
2.0Kb
d
Enrichment Plot:
ERα/TEAD Co-binding； ERα target Gene
Enrichment score (ES)
NES=-2.831
NOM p-val< 0.001
e
Enrichment Plot:
ERα/TEAD Co-binding ERα target Gene；
NES=-2.026
NOM p-val< 0.001
f
139/165
(84%)
26/165
(16%)
ERα/TEAD co-binding genes
ERα only binding genes
g
ERα
TEAD1
YAP
H3K27Ac
hg19
Greb1
ESR1
CCND1
PDZK1
PS2
MYOF
SLC9A3R1
PGR
SEC14L2
RAPGEFL1
TH
CYR61
CTGF

Supplementary Fig. 6 Global analysis of ERα, TEAD, and YAP co-binding

a, Venn diagram showing the numbers of ERα binding peaks overlapping with TEAD1 and YAP ChIP-seq binding peaks in MCF-7 cells.
b, Heatmaps of ERα, YAP, TEAD1 and H3K27Ac ChIP-Seq reads from MCF-7 cells showing the bound loci of $ER\alpha^{+}$/$TEAD1^{+}$/YAP^{+}, $ER\alpha^{+}$/$TEAD1^{+}$/YAP^{-} and $ER\alpha^{-}$/$TEAD1^{+}$/YAP^{+}.
c, Average tag density plots of H3K27Ac at indicated gene binding sites within 2kb frames in MCF-7 cells.
d, Gene set enrichment analysis (GSEA) for the indicated gene set in RNA-Sequencing from XMU-MP-1 treated MCF-7 cells.
e, GSEA for the indicated gene set in RNA-Sequencing from YAP-5SA overexpression MCF-7 cells.
f, Among the 165 XMU-MP-1 downregulated ERα target genes that contain ERα binding peaks in their promoter/enhancer regions, cells, 139 contain ERα/TEAD1 co-binding peaks whereas 26 contain ERα only binding peaks.
g, ChIP-seq signal tracks of ERα, TEAD1, YAP and H3K27Ac at promoter/enhancer regions of the indicated ERα target genes downregulated by XMU-XP-1 in MCF-7 cells. *GREB1*, *ESR1*, *CCND1*, *PDZK1*, *PS2*, *MYOF*, *SLC9A3R1* and *PGR* contain ERα/TEAD co-binding peaks whereas *SEC14L2* and *TH* contain ER only binding peaks.

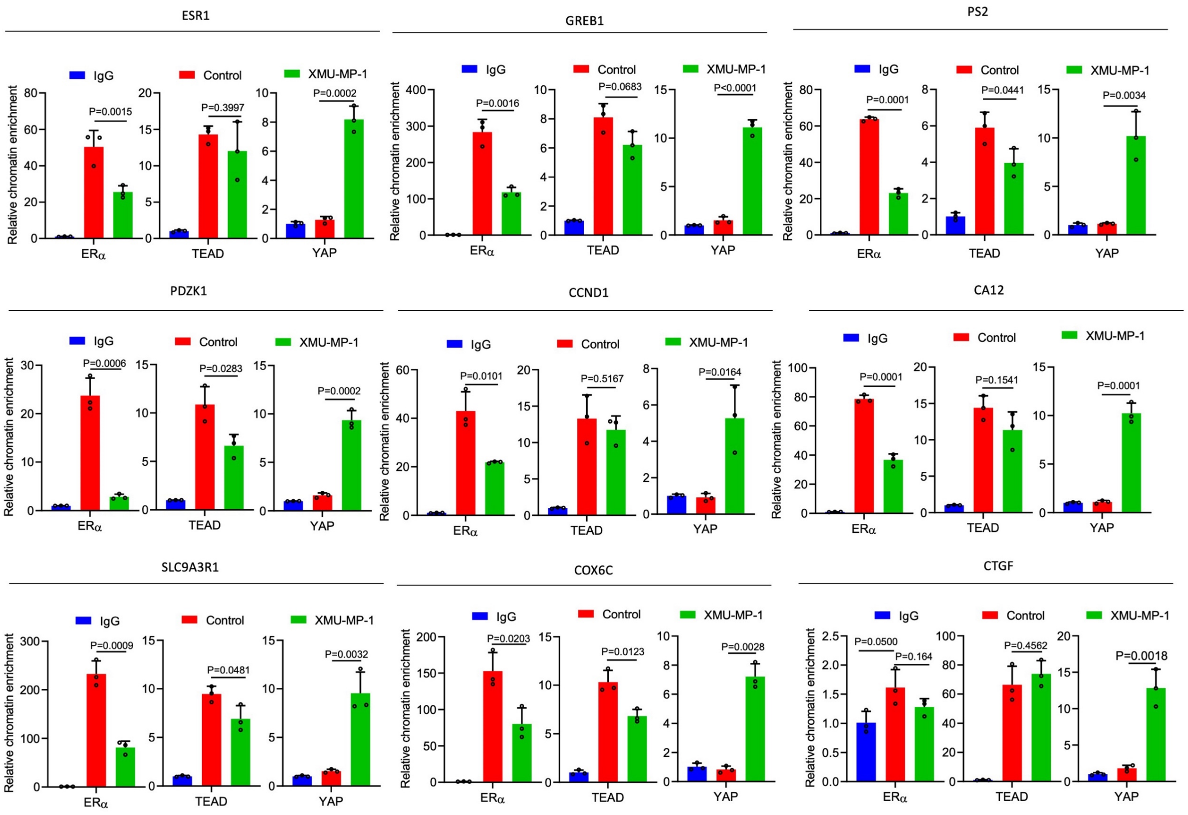

Supplementary Fig. 7 ChIP analysis of chromatin binding of ERα/TEAD/Yap

XUM-MP-1 increased the promoter/enhancer occupancy of YAP while reduced the promoter/enhancer occupancy of ERα to the indicated ERα target genes including *ESR1*. MCF-7 cells were treated with 3 μM XMU-MP-1 for 6 hours, followed by ChIP qPCR analysis for ERα, TEAD4, YAP binding to the promoter/enhancer regions of the indicated ERα target genes as well as the YAP target gene *CTGF*.
Results are representative of 3 independent experiments. Data are means ± s.d. Two-sided, unpaired t-test were used for this figure. Source data are provided in the Source Data file.

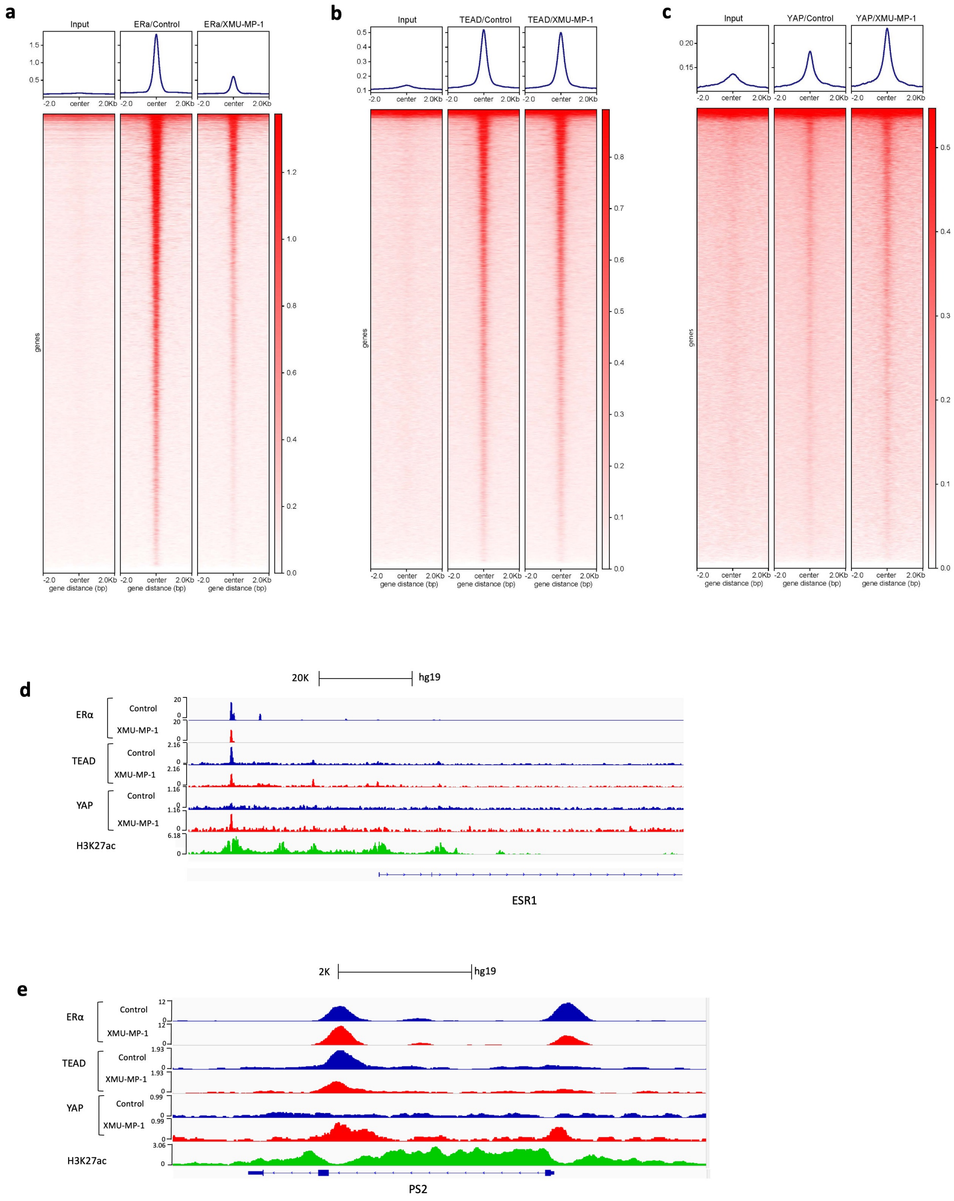

Supplementary Fig. 8 ChIP-seq analysis of chromatin binding of ERα/TEAD/YAP

a, b, c, Heatmaps of ERα (**a**) TEAD (**b**) and YAP (**c**) ChIP-Seq reads from MCF-7 cells treated with vehicle (control) or XMU-MP-1 showing the bound loci of ERα/TEAD sites
d, e, ChIP-seq signal tracks of ERα, TEAD, YAP and H3K27Ac at promoter/enhancer regions of the indicated ERα target genes.

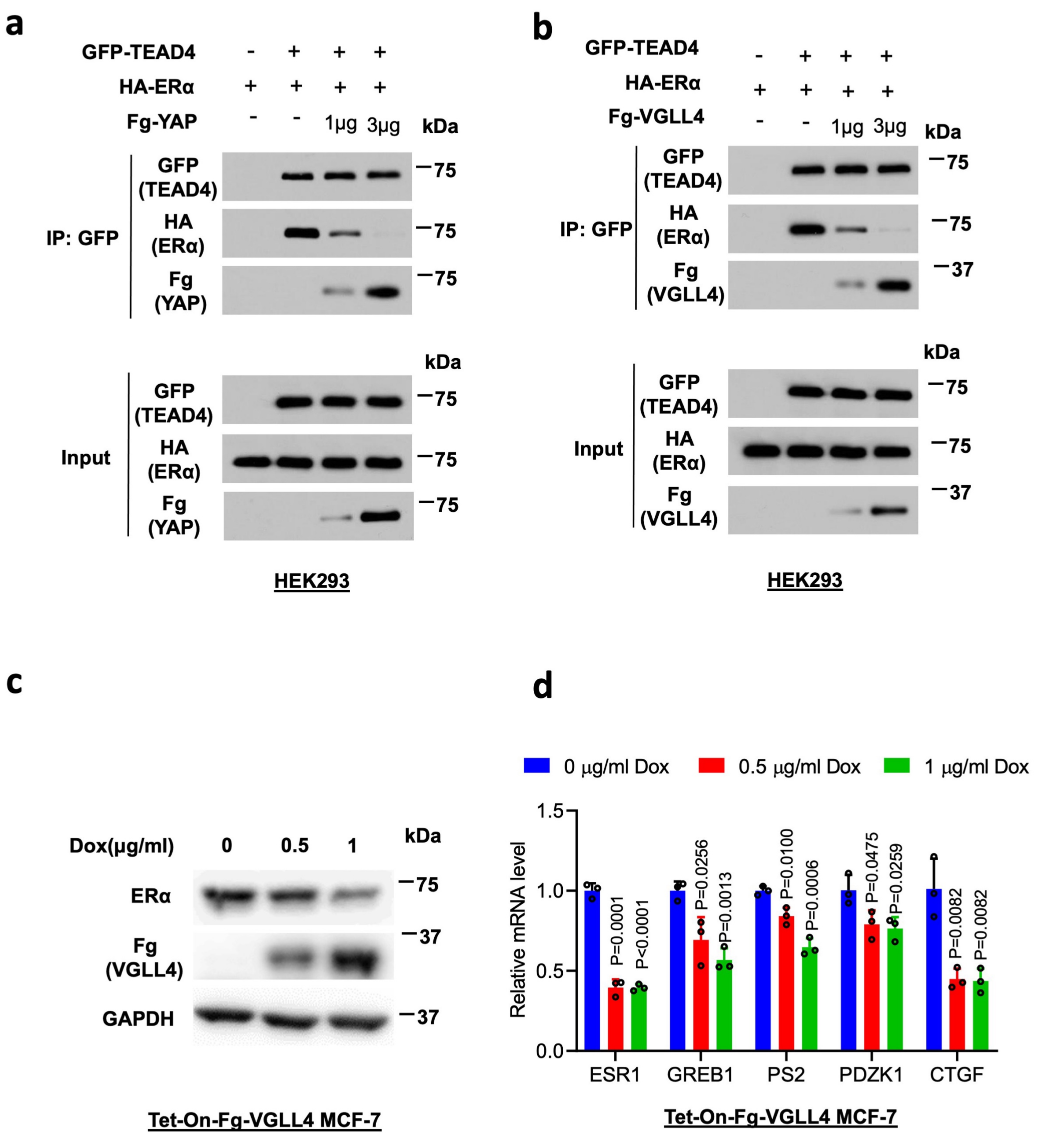

Supplementary Fig. 9 VGLL4 inhibits ERα by competing for TEAD binding

a, b, Coexpression of YAP or VGLL4 with ERα and TEAD4 disrupted their association. HEK293T cells were transfected with fixed amounts of GFP-TEAD4 and HA- ERα and increasing amounts of Flag-YAP (**a**) or Flag-VGLL4 (**b**), followed by Co-IP and western blot analyses with the indicated antibodies.

c, d, Induced expression of VGLL4 blocked ERα transcriptional activity. MCF-7 cells expressing Tet-O-VGLL4 were treated without or with doxycycline (Dox) at the indicated concentration, followed by western blot analysis for Fg-VGLL4 and ERα (**c**) and RT-qPCR analysis for ERα target gene expression (**d**).

Results shown in **a-d** are representative of 3 independent experiments. Data are means ± s.d. Two-sided, unpaired t-test for **d**. Source data are provided in the Source Data file.

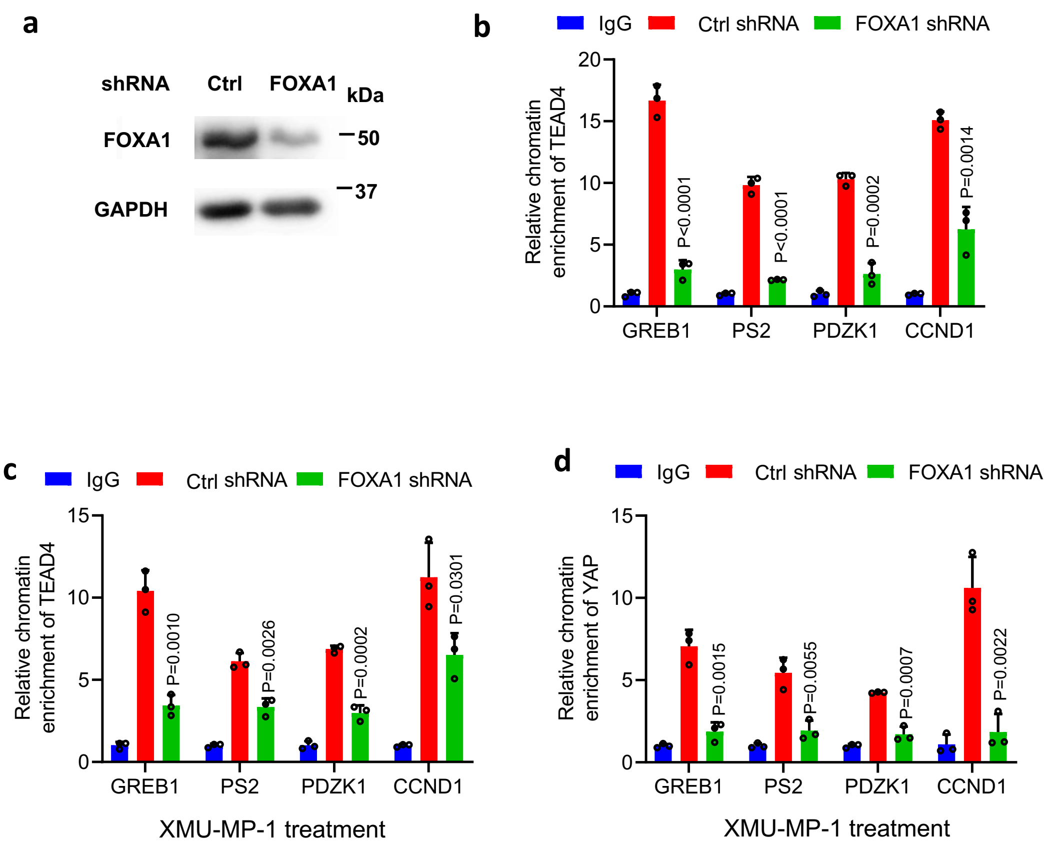

Supplementary Fig. 10 FOXA1 is required for YAP/TEAD binding to ERα target loci

a, Western blot analysis of FOXA1 expression in MCF-7 transfected with control (Ctrl) or FOXA1 shRNA
b, ChIP qPCR assay showed that FOXA1 RNAi decreased TEAD4 recruitment to the indicated ERα target gene promoters/enhancers in MCF-7 cells.
c, d, ChIP qPCR assay showed that FOXA1 RNAi decreased TEAD4 (**c**) or YAP (**d**) recruitment to the indicated ERα target gene promoters/enhancers in MCF-7 cells treated with XMU-MP-1.
Results shown in **a-d** are representative of 3 independent experiments. Data are means ± s.d. Two-sided, unpaired t-test for **b-d**. Source data are provided in the Source Data file.

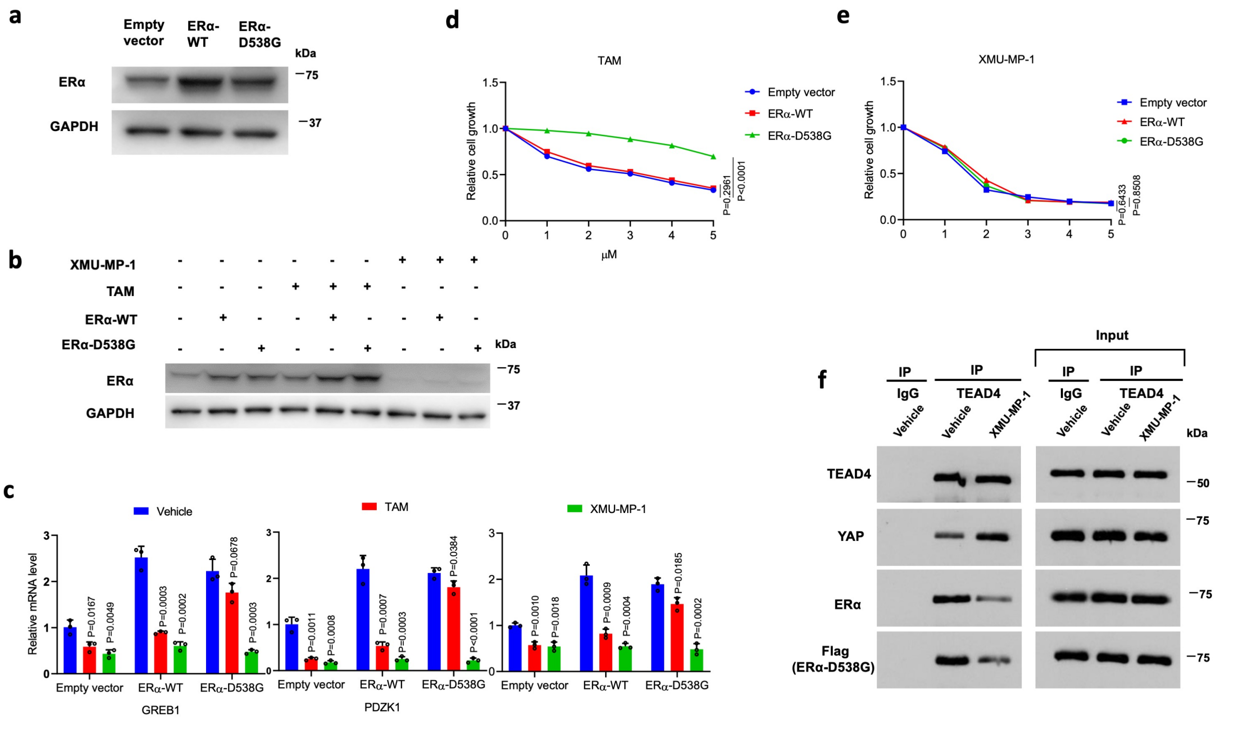

Supplementary Fig. 11 XMU-MP-1 inhibits D538G ERα mutant

a, Western blot analysis to show the expression ERα in control and MCF-7 cells transfected with lentivirus carrying wild type (WT) or mutant (D538G) ERα.
b, XMU-MP-1 downregulated both ERα-WT and ERα-D538G. MCF-7 cells expressing ERα-WT or ERα-D538G were treated with 3 μM XMU-MP-1 or 1 μM tamoxifen for 24 hours, followed by western blot analysis. Results are representative of two independent experiments.
c, XMU-MP-1 but not tamoxifen (TAM) suppressed ERα-D538G activity. Control and MCF-7 cells expressing ERα-WT or ERα-D538G were treated with 3 μM XMU-MP-1 or 1 μM tamoxifen for 24 hours, followed by RT-qPCR analysis of ERα target gene expression. Results are representative of 3 independent experiments.
d,e, XMU-MP-1 but not tamoxifen inhibited the growth of ERα-D538G expressing cells. MCF-7 cells expressing ERα-WT or ERα-D538G were seeded into 96-well plates and treated with tamoxifen (**d**) or XMU-MP-1 (**e**) at the indicated concentrations, followed by the WST-1 assay. Results are representative of two independent experiments.
f, XMU-MP-1 inhibited TEAD4/ERα-D538G interaction. MCF-7 cells expressing ERα-D538G were treated with 3 μM XMU-MP-1 for 2 hours, followed by Co-IP and western blot analysis with the indicated antibodies. Results are representative of 2 independent experiments.
Data are means ± s.d. Two-sided, unpaired t-test, were used for **c**. Source data are provided in the Source Data file
