# Supplementary material for: YAP inhibits ERα and ER+ breast cancer growth by disrupting a TEAD-ERα signaling axis
Source: Nat Commun. 2022 Jun 2;13:3075. doi: 10.1038/s41467-022-30831-5 (PMC9163075; doi:10.1038/s41467-022-30831-5)
Supplement: Supplementary file 2 — Description of Additional Supplementary Files [file 41467_2022_30831_MOESM2_ESM.pdf]

Description of Additional Supplementary Files

Supplementary Data 1

Pathology and subtype of tumors used in Fig. 1g

Supplementary Data 2

Pathology and subtype of tumors used in Fig. 1h

Supplementary Data 3

List of XMU-MP-1 downregulated ERα target genes that contain ERα/TEAD co-binding peaks or ER only binding peaks.
