# Supplementary material for: YAP inhibits ERα and ER+ breast cancer growth by disrupting a TEAD-ERα signaling axis
Source: Nat Commun. 2022 Jun 2;13:3075. doi: 10.1038/s41467-022-30831-5 (PMC9163075; doi:10.1038/s41467-022-30831-5)
Supplement: Supplementary file 6 — Reporting Summary [file 41467_2022_30831_MOESM6_ESM.pdf]

nature portfolio

Corresponding author(s): Dr. Jin Jiang and Dr. Jian Zhu

Last updated by author(s): Aug 24, 2021

Reporting Summary

Nature Portfolio wishes to improve the reproducibility of the work that we publish. This form provides structure for consistency and transparency in reporting. For further information on Nature Portfolio policies, see our Editorial Policies and the Editorial Policy Checklist.

Statistics

For all statistical analyses, confirm that the following items are present in the figure legend, table legend, main text, or Methods section.

n/a	Confirmed	
☐	☒	The exact sample size (*n*) for each experimental group/condition, given as a discrete number and unit of measurement
☐	☒	A statement on whether measurements were taken from distinct samples or whether the same sample was measured repeatedly
☐	☒	The statistical test(s) used AND whether they are one- or two-sided<br>*Only common tests should be described solely by name; describe more complex techniques in the Methods section.*
☒	☐	A description of all covariates tested
☒	☐	A description of any assumptions or corrections, such as tests of normality and adjustment for multiple comparisons
☐	☒	A full description of the statistical parameters including central tendency (e.g. means) or other basic estimates (e.g. regression coefficient) AND variation (e.g. standard deviation) or associated estimates of uncertainty (e.g. confidence intervals)
☐	☒	For null hypothesis testing, the test statistic (e.g. *F*, *t*, *r*) with confidence intervals, effect sizes, degrees of freedom and *P* value noted<br>*Give P values as exact values whenever suitable.*
☒	☐	For Bayesian analysis, information on the choice of priors and Markov chain Monte Carlo settings
☒	☐	For hierarchical and complex designs, identification of the appropriate level for tests and full reporting of outcomes
☒	☐	Estimates of effect sizes (e.g. Cohen's *d*, Pearson's *r*), indicating how they were calculated

Our web collection on statistics for biologists contains articles on many of the points above.

Software and code

Policy information about availability of computer code

Data collection	The RNA-seq data were deposited in the Gene Expression Omnibus (GEO) database (Assessing number: GSE165288). ChIP-seq data were deposited in the GEO database (Assessing number: GSE197239), we also retrieved from GEO under accession GSE72249 and GSE107013.
Data analysis	For ChIP-seq data, SRA files were downloaded for re-analysis. Reads were aligned to the human genome (hg19) using 'Bowtie2'(Version 2.4.4), reads were sorted using 'Samtools" (Version 1.15.1)subsequently. Peaks were identified using MACS2 (v2.1.1) with the p-value cutoff 1e-5. For RNA seq data, Analysis was performed for differentially expressed genes (P < 0.01 and fold change > 2) by Ingenuity Pathway Analysis (IPA).For gene set enrichment analysis of RNA-seq data, gene sets of Hallmark Estrogen Response Early and Cordenonsi YAP Conserved Signature were used and downloaded from Molecular Signatures Database v7.4, GSEA was implemented using the GSEA 4.1.0 software, with default parameters.

For manuscripts utilizing custom algorithms or software that are central to the research but not yet described in published literature, software must be made available to editors and reviewers. We strongly encourage code deposition in a community repository (e.g. GitHub). See the Nature Portfolio guidelines for submitting code & software for further information.

Data

Policy information about availability of data

All manuscripts must include a data availability statement. This statement should provide the following information, where applicable:

- Accession codes, unique identifiers, or web links for publicly available datasets
- A description of any restrictions on data availability
- For clinical datasets or third party data, please ensure that the statement adheres to our policy

The data of Figs. 1c-e and Supplementary Figs. 1a-d are available at TCGA website, gene expression data for 1218 TCGA breast cancer patients were downloaded

from the webpage (http://xena.ucsc.edu/). The Data of Supplementary Fig.1i and j are available at METABRIC database website, METABRIC breast cancer data were downloaded from cbioportal (https://www.cbioportal.org/). The RNA-seq data are available in the GEO database with accession number GSE165288 and GSE107010, the ChIP-seq data are available in the GEO database with accession number, GSE197239, GSE72249 and GSE107013.

Field-specific reporting

Please select the one below that is the best fit for your research. If you are not sure, read the appropriate sections before making your selection.

☒ Life sciences ☐ Behavioural & social sciences ☐ Ecological, evolutionary & environmental sciences

For a reference copy of the document with all sections, see nature.com/documents/nr-reporting-summary-flat.pdf

Life sciences study design

All studies must disclose on these points even when the disclosure is negative.

Sample size	For cell line-based experiments, at least three independent samples for each group for statistical calculations. For clinical data analysis, one hundred and forty two breast cancer samples were analyzed for IHC analysis.
Data exclusions	No data were excluded from analysis
Replication	For most experiment, at least 3 biological repeats were done. For Fig1g, Fig2f-h, Fig7 g-m were repeat once. Fig1g is based on a lot of patient samples analysis, and for the others, to minimize the amount mouses used,and increase the results quality, we used 7 repeat samples for each group.
Randomization	Before the experiments, all the samples were grouped randomly for each participant, and repeat these results at different time.
Blinding	All the experiments were double blinded. One of the participant prepare samples, another one perform the experiments, finally all participants analysis the results together.

Reporting for specific materials, systems and methods

We require information from authors about some types of materials, experimental systems and methods used in many studies. Here, indicate whether each material, system or method listed is relevant to your study. If you are not sure if a list item applies to your research, read the appropriate section before selecting a response.

Materials & experimental systems

n/a	Involved in the study	
☐	☒	Antibodies
☐	☒	Eukaryotic cell lines
☒	☐	Palaeontology and archaeology
☐	☒	Animals and other organisms
☐	☒	Human research participants
☒	☐	Clinical data
☒	☐	Dual use research of concern

Methods

n/a	Involved in the study	
☐	☒	ChIP-seq
☒	☐	Flow cytometry
☒	☐	MRI-based neuroimaging

Antibodies

Antibodies used	For Immunoblot analysis: Anti- YAP (Santa Cruz, Cat. No. SC-101199); Anti- ERα (Cell Signaling Technology, Cat. No. SC-D8H8); Anti-TEAD4 (Santa Cruz, Cat. No. Sc-390578); Anti-TEAD1(BD Transduction Laboratories, Cat. No. 610922), for Co-IP, ERα (Santa Cruz, Cat. No. SC8005), YAP (Santa Cruz, Cat. No. SC-101199), or TEAD4 (Santa Cruz, Cat. No. Sc-390578) , for Immunofluorescence assay, anti-ERα polyclonal antibody (Cell Signaling Technology, Cat. No. 8644S) and mouse anti-YAP monoclonal antibodies (Santa Cruz, Cat. No. SC-101199) , for ChIP, anti-ERα (Santa Cruz, Cat. No. sc-8002x), anti-TEAD4 (Santa Cruz, Cat. No. sc-101184), and anti-YAP (Santa Cruz, Cat. No. sc-271134)
Validation	Anti- YAP (Santa Cruz, Cat. No. SC-101199),mouse monoclonal antibody raised against recombinant YAP of human origin. validated by WB and Immunofluorescence staining.<br>Anti-YAPSanta Cruz, Cat. No. SC-271134), mouse anti human, validated by WB and Immunofluorescence staining.<br>Anti-TEAD4 (Santa Cruz, Cat. No. Sc-390578), mouse anti human, validated by WB.<br>anti-TEAD4 (Santa Cruz, Cat. No. sc-101184), mouse anti human, validated by WB.<br>Anti-TEAD1(BD Transduction Laboratories, Cat. No. 610922), mouse anti human, validated by WB and Immunofluorescence staining.<br>Anti-ERα (Santa Cruz, Cat. No. SC8005), mouse anti human, validated by WB and Immunofluorescence staining.<br>Anti-ERα (Santa Cruz, Cat. No. SC8002x), mouse anti human, validated by WB and Immunofluorescence staining.<br>Anti- ERα (Cell Signaling Technology, Cat. No. SC-D8H8), Rabbit anti human, validated by ChIP, WB and C&R.<br>ERα polyclonal antibody (Cell Signaling Technology, Cat. No. 8644S), Rabbit anti human, validated by ChIP, WB and C&R.

Eukaryotic cell lines

Policy information about cell lines

Cell line source(s)	MCF-7, T47D, MDA-MB-231 and HEK293T cells are acquired form American Type Culture Collection (ATCC)
Authentication	Cell lines were subject to STR authentication
Mycoplasma contamination	We tested mycoplasma to make sure there is no contamination.
Commonly misidentified lines (See ICLAC register)	no commonly misidentified cell lines were used in the study

Animals and other organisms

Policy information about studies involving animals; ARRIVE guidelines recommended for reporting animal research

Laboratory animals	All mice were kept under specific pathogen free (SPF) and temperature-controlled environment. Housing condition for mice: 20±2°C, 50±5% humidity, 12h-12h light-dark cycles. Female 6-week-old NOD scid gamma (NSG, NOD.Cg-Prkdc(scid)Il2rg(tm1Wji)/SzJ strain, The Jackson Laboratory, Maine, USA) mice were used for experiments
Wild animals	no wild animals were used in the study
Field-collected samples	no field collected samples
Ethics oversight	The procedures for all animal experiments were reviewed and approved by the IACUC of UT Southwestern Medical School.

Note that full information on the approval of the study protocol must also be provided in the manuscript.

Human research participants

Policy information about studies involving human research participants

Population characteristics	We selected discarded surgical samples after patient provided informed consent. We select ER positive and ER negative tumor samples in this study. We didn't publish any identifiable images or information in this study.
Recruitment	We select ER positive and ER negative tumor samples in this study.
Ethics oversight	This study was reviewed and approved by the Ethical Board at the Qilu Hospital of Shandong University with written informed consent from all the patients.

Note that full information on the approval of the study protocol must also be provided in the manuscript.

ChIP-seq

Data deposition

☒ Confirm that both raw and final processed data have been deposited in a public database such as GEO.

☒ Confirm that you have deposited or provided access to graph files (e.g. BED files) for the called peaks.

Data access links *May remain private before publication.*	ChIP-seq data were deposited in the GEO database (Assessing number: GSE197239), And We also used public available data, while the GEO numbers were provided in the methods section.
Files in database submission	We submitted raw data and Bigwig files to GEO database. We also used public available data, while the GEO numbers were provided in the methods section
Genome browser session (e.g. UCSC)	Reads were aligned to the human genome (hg19) using 'Bowtie2', reads were sorted using 'Samtools" subsequently.

Methodology

Replicates	We used public available data, while the GEO numbers were provided in the methods section. We also repeat the ChIP-RT PCR at least 3 times to validate the results.
Sequencing depth	The reads number of ChIP-seq is 15M-25M. And we also used public available data, while the GEO numbers were provided in the methods section
Antibodies	anti-ERα (Santa Cruz, Cat. No. sc-8002x), anti-TEAD4 (Santa Cruz, Cat. No. sc-101184), and anti-YAP (Santa Cruz, Cat. No. sc-271134)

Peak calling parameters	Peaks were identified using MACS2 v2.1.1 with the p-value cutoff 1e-5. After peak calling, ENCODE human blacklisted regions were removed from the peak files subsequently. ChIP-seq signal tracks were generated by 'DeepTools' and normalized by RPKM. Signal tracks were visualized by Integrative Genomics Viewer (IGV) software.
Data quality	The ChIP-seq data were assessed by FASTQC, and We also used public available data, while the GEO numbers were provided in the methods section.
Software	Peak overlapping analysis and subsets annotation were performed using 'HOMER' v4.9. Heatmaps and signal plots for the ChIP-seq peak subsets were generated using 'DeepTools'.
